# Effect of inferior caval valve implantation on circulating immune cells and inflammatory mediators in severe tricuspid regurgitation

**DOI:** 10.1186/s12872-024-04044-1

**Published:** 2024-07-18

**Authors:** Isabel Mattig, Bernd Hewing, Fabian Knebel, Christian Meisel, Antje Ludwig, Frank Konietschke, Verena Stangl, Karl Stangl, Michael Laule, Henryk Dreger

**Affiliations:** 1 Department of Cardiology, Angiology and Intensive Care Medicine, Deutsches Herzzentrum der Charité, Campus Virchow-Klinikum Augustenburger Platz 1, Berlin, 13353 Germany; 2 Charité – Universitätsmedizin Berlin, Corporate Member of Freie Universität Berlin and Humboldt-Universität zu Berlin, Charitéplatz 1, Berlin, 10117 Germany; 3 Berlin Institute of Health at Charité – Universitätsmedizin Berlin, BIH Biomedical Innovation Academy, Berlin, Germany; 4 Partner Site Berlin, DZHK (German Centre for Cardiovascular Research), Berlin, Germany; 5 Sana Klinikum Lichtenberg, Innere Medizin II: Schwerpunkt Kardiologie, Berlin, Germany; 6 Department of Cardiology III - Adult Congenital and Valvular Heart Disease, University Hospital Muenster, Muenster, Germany; 7 Labor Berlin – Charité Vivantes Services GmbH, Berlin, Germany; 8 Institute for Biometry and Clinical Epidemiology, Charité – Universitätsmedizin Berlin, corporate member of Freie Universität Berlin and Humboldt-Universität zu Berlin, Charitéplatz 1, Berlin, 10117 Germany; 9 Department of Cardiology, Angiology and Intensive Care Medicine, Deutsches Herzzentrum der Charité, Campus Charité Mitte, Charitéplatz 1, Berlin, 10117 Germany

**Keywords:** Tricuspid regurgitation, Inferior vena cava, Valve implantation, Immune status, Immune system

## Abstract

**Background:**

Interventional valve implantation into the inferior vena cava (CAVI) lowers venous congestion in patients with tricuspid regurgitation (TR). We evaluated the impact of a reduction of abdominal venous congestion following CAVI on circulating immune cells and inflammatory mediators.

**Methods:**

Patients with severe TR were randomized to optimal medical therapy (OMT) + CAVI (*n* = 8) or OMT (*n* = 10). In the OMT + CAVI group, an Edwards Sapien XT valve was implanted into the inferior vena cava. Immune cells and inflammatory mediators were measured in the peripheral blood at baseline and three-month follow-up.

**Results:**

Leukocytes, monocytes, basophils, eosinophils, neutrophils, lymphocytes, B, T and natural killer cells and inflammatory markers (C-reactive protein, interferon-gamma, interleukin-2, -4, -5, -10, and tumor necrosis factor-alpha) did not change substantially between baseline and three-month follow-up within the OMT + CAVI and OMT group.

**Conclusion:**

The present data suggest that reduction of venous congestion following OMT + CAVI may not lead to substantial changes in systemic inflammation within a short-term follow-up.

**Clinical trial registration:**

NCT02387697

**Graphical Abstract:**

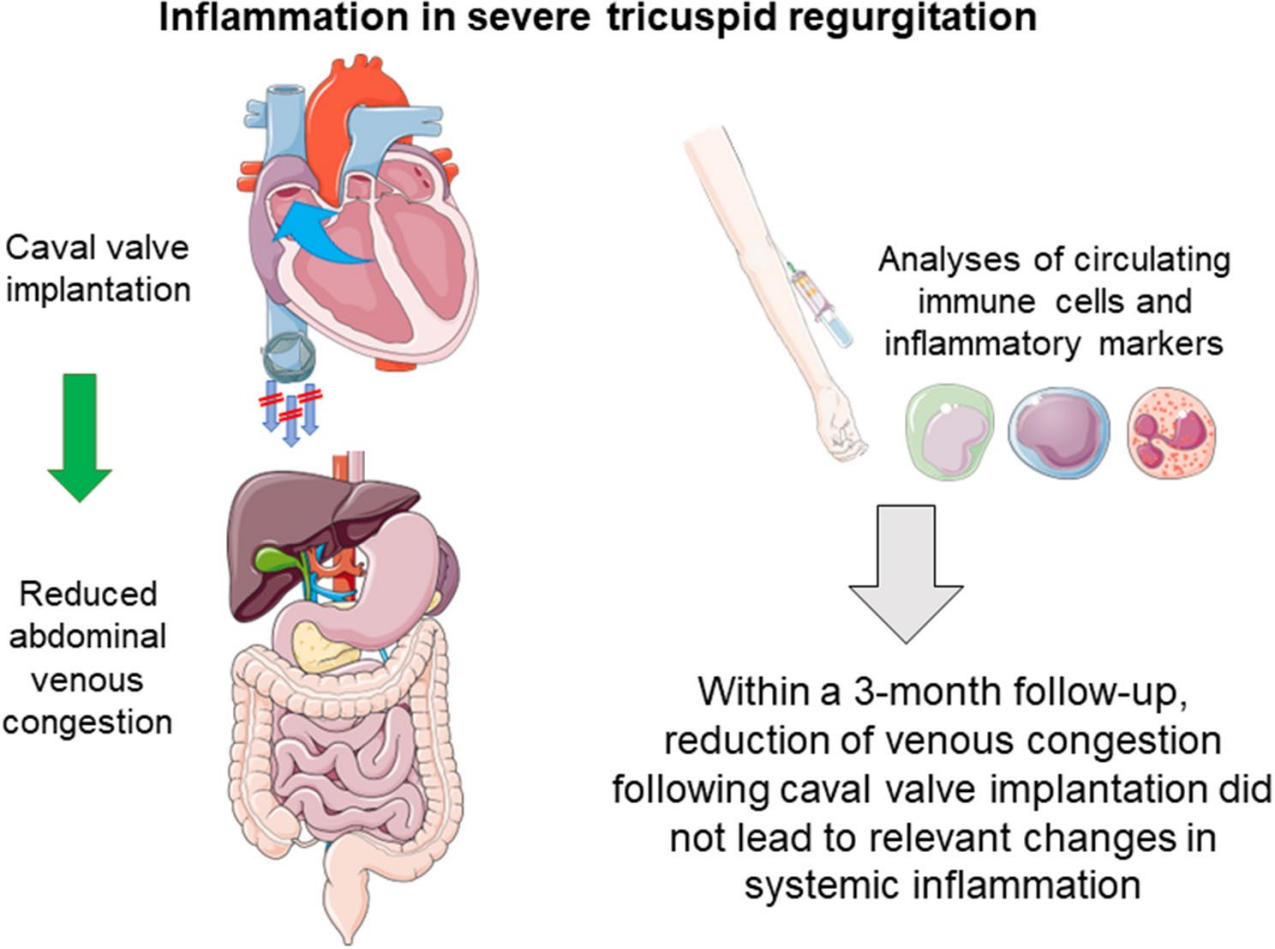

**Supplementary Information:**

The online version contains supplementary material available at 10.1186/s12872-024-04044-1.

## Background

Tricuspid regurgitation (TR) is a determinant for reduced functional capacity and survival [1, 2]. It frequently occurs in elderly patients resulting in right heart failure with progressive venous congestion [1, 3]. Venous congestion causes the release of inflammatory markers [4–6] and circulating cell adhesion molecules [7]. These inflammatory processes may lead to adverse remodelling and malfunction of the right heart due to oxidative stress, cell death, fibrosis, alteration of extracellular matrix and of cellular metabolism [8, 9]. In addition, severe valvular heart disease such as aortic stenosis is associated with changes in the amount of circulating immune cells, e.g., intermediate monocytes [10]. Treatment of both tricuspid regurgitation and the reduction of venous congestion may attenuate systemic inflammation and thereby, mitigate deterioration of right heart function. Therefore, we hypothesized, that transfemoral implantation of a heart valve (CAVI) into the inferior vena cava (IVC) combined with optimal medical therapy results in a decrease of systemic inflammation due to a reduction of abdominal venous congestion.

In the present subanalysis of the TRICAVAL study, we aimed to evaluate the impact of a reduction of abdominal venous congestion following CAVI on circulating immune cells and inflammatory mediators.

## Methods

### Study design

As previously published, the TRICAVAL study (Treatment of Severe Secondary Tricuspid Regurgitation in Patients with Advance Heart Failure with Caval Vein Implantation of the Edwards Sapien XT Valve, NCT02387697) is an investigator initiated, single-centre trial [11]. The study was approved by local ethics committee (Landesamt für Gesundheit und Soziales Berlin, Berlin, Germany) and state authorities (Bundesinstitut für Arzneimittel und Medizinprodukte, Bonn, Germany) [11]. All participants provided written informed consent [11]. After screening of 87 patients, 28 patients were randomized to optimal medical therapy (OMT) + CAVI (*n* = 14) or OMT (*n* = 14) [11]. Inclusion criteria comprised severe, symptomatic TR despite optimal medical therapy and a high surgical risk or contraindications for surgery [11]. Detailed inclusion and exclusion criteria were reported by Dreger et al. [11]. In the OMT + CAVI group, an Edwards Sapien XT valve (Edwards Lifesciences, Irvine, CA, USA) was implanted via transfemoral access into the IVC after preparation with two or three self-expandable stents (sinus XL, Optimed, Ettlingen, Germany) [11]. OMT consisted of heart failure medication including diuretic therapy, beta blockers, angiotensin converting enzyme inhibitors or angiotensin II receptor blocker and mineralocorticoid receptor antagonists, depending on heart failure classification and symptoms [11]. Immune cells and mediators were evaluated in blood drawn from a peripheral vein of the upper extremity at baseline and three-month follow-up. Due to safety concerns (six deaths in the OMT + CAVI group and one death in the OMT group), the study was stopped prematurely after two valve dislocations and two stent migrations occurred [11, 12]. Three patients in the OMT group withdrew consent within the three-month follow-up. Thus, immune status was analyzed in 10 patients in the OMT and 8 patients in the OMT + CAVI group. The tables in the results section of this manuscript provide the actual numbers of patients examined for each individual parameter.

### Measurements of immune cells and inflammatory mediators

Standard white blood cell differential was performed using the XE-5000 Case Manager hematology analyzer (Sysmex, Norderstedt, Germany). C-reactive protein (CRP) was determined using a latex-enhanced turbidimetric immunoassay on a Cobas 8000 analyzer (Roche Diagnostics, Mannheim, Germany).

Enumeration and phenotyping of lymphocyte subsets was performed in EDTA whole blood samples within 4 h after blood collection using accredited test methods (DIN EN ISO 15,189) at the Department of Immunology at Labor Berlin, as described previously [13, 14]. Briefly, the following mouse anti-human fluorescently-labelled monoclonal antibodies (all from Beckman Coulter, Krefeld, Germany) were used for quantification of lymphocytes subsets, naive/memory T cell subsets, regulatory T cells and analysis of T cell activation markers: cluster of differentiation (CD)3 Allophycocyanine-Alexa Fluor 750 (APC-A750, clone UCHT1; catalog number A94680), CD4 energy coupled dye (ECD, clone SCFI12T4D11; 6,604,727), CD8 APC (clone B9.11; IM2469), CD11a Fluorescein isothiocyanate (FITC, clone 25.3; IM0860U) CD14 FITC (clone RMO52; B36297), CD16 Phycoerythrine (PE, clone 3G8; A07766), CD19 PE-Cy5.5 (clone J3-119; B49211), CD45RA Pacific-Blue (PB, clone J33; A74763), CD56 PE (clone N901; A07788), CD57 PB (clone NC1; A74779), HLA-DR PE (clone Immu-357; IM1639) and CCR7 PE (clone G043H7; B30632). Stained samples were acquired on a ten-colour Navios flow cytometer and analyzed using Navios Software (Beckman Coulter).

Concanavalin (Con) A-induced lymphocytic interferon-gamma (IFN-γ), tumor necrosis factor-alpha (TNF-α), interleukin (IL)-2, IL-4, IL-5 and IL-10 secretion was analyzed in 24 h stimulated heparinized whole blood sample supernatants by Cytometric Bead Array (BD Biosciences, Heidelberg, Germany), as described [15].

### Statistical analysis

Data from all patients with completed three-month follow-up (OMT + CAVI *n* = 8, OMT *n* = 10) were analyzed by SPSS Statistics version 28 (IBM Corporation, New York, NY, USA). Continuous variables are presented as median with 25th and 75th percentile for a better comparison. Statistical analysis was performed using Wilcoxon test for intragroup comparison. A *p*-value of < 0.05 was defined as statistically significant and z values were added to present effects over time.

## Results

Twenty-eight patients with severe TR were randomized to OMT + CAVI (*n* = 14) or OMT group (*n* = 14); eighteen patients completed the three-month follow-up. Baseline characteristics of the study participants are listed in Table 1. None of the patients had a history of stroke, peripheral artery disease, bronchial asthma, or dialysis. None of the patients had signs of an acute infection based on clinical status and laboratory. In the OMT group, one patient suffered from an active cancer disease (chronic myelogenous leukaemia) and one patient from monoclonal gammopathy of undetermined significance. No relevant autoimmune diseases were reported in the OMT + CAVI group. Echocardiographic examinations revealed a significant reduction of abdominal-venous congestion after OMT + CAVI at three-month follow-up [16]. Medication including heart failure therapy and pharmacological treatment with potential effect on levels of inflammatory markers at baseline and at three-month follow-up are listed in Table 2.

**Table 1:** Baseline characteristics

	OMT (*n* = 10)	OMT + CAVI (*n* = 8)
Female, n (%)	5 (50)	6 (75)
Age, years (IQR)	78 (73.3–83.9)	79 (68.3–82.6)
BMI, kg/m² (IQR)	25.0 (21.4–27.4)	27.1 (24.9–30.4)
LVEF, % (IQR)	60.0 (54.3–61.3)	60.0 (52.5–62.0)
TAPSE, mm (IQR)	15.0 (11.8–22.0)	16.5 (13.3–18.0)
NT-proBNP, ng/l (IQR)	2233.0 (1596.3–3954.0)	2342.0 (1404.8–2740.3)
Coronary artery disease, n (%)	5 (50)	3 (38)
Arterial hypertension, n (%)	8 (80.0)	7 (87.5)
Diabetes mellitus, n (%)	4 (40.0)	3 (37.5)
Active cancer, n (%)	1 [10]	0 (0)
History of cancer, n (%)	2 [20]	1 [13]
Smoking status, n (%)	0 (0)	0 (0)

Continuous variables are shown as median and interquartile ranges, categorical variables are given as absolute number with percentages. OMT, optimal medical therapy; CAVI, caval valve implantation; NYHA class, New York Heart Association Class; BMI, body mass index; LVEF, left ventricular ejection fraction; TAPSE, tricuspid annular plane systolic excursion; NT-proBNP, N-terminal pro brain natriuretic peptide; ACE, angiotensin-converting enzyme

**Table 2:** Medication at baseline and three-month follow-up

	OMT (*n* = 10)		OMT + CAVI (*n* = 8)	
	Baseline	3 months	Baseline	3 months
ACE inhibitor, n (%)	6 (60)	6 (60)	7 (88)	6 (75)
Beta-blocker, n (%)	9 (90)	8 (80)	8 (100)	7 (88)
Mineralocorticoid receptor antagonist, n (%)	6 (60)	6 (60)	5 (63)	4 (50)
SGLT-II-inhibitors, n (%)	0 (0)	0 (0)	0 (0)	0 (0)
Diuretics, n (%)	10 (100)	10 (100)	8 (100)	7 (88)
Statin Therapy, n (%)	6 (60)	4 (40)	5 (62.5)	5 (63)
Xanthine oxidase inhibitor, n (%)	3 [30]	4 (40)	3 (38)	2 [25]

Continuous variables are shown as median and interquartile ranges due to the distribution of parameters (uniform per variable). OMT, optimal medical therapy; CAVI, caval valve implantation; *n*, number of patients with laboratory measurements in case of missing data

### Circulating immune cells

**Fig. 1:**
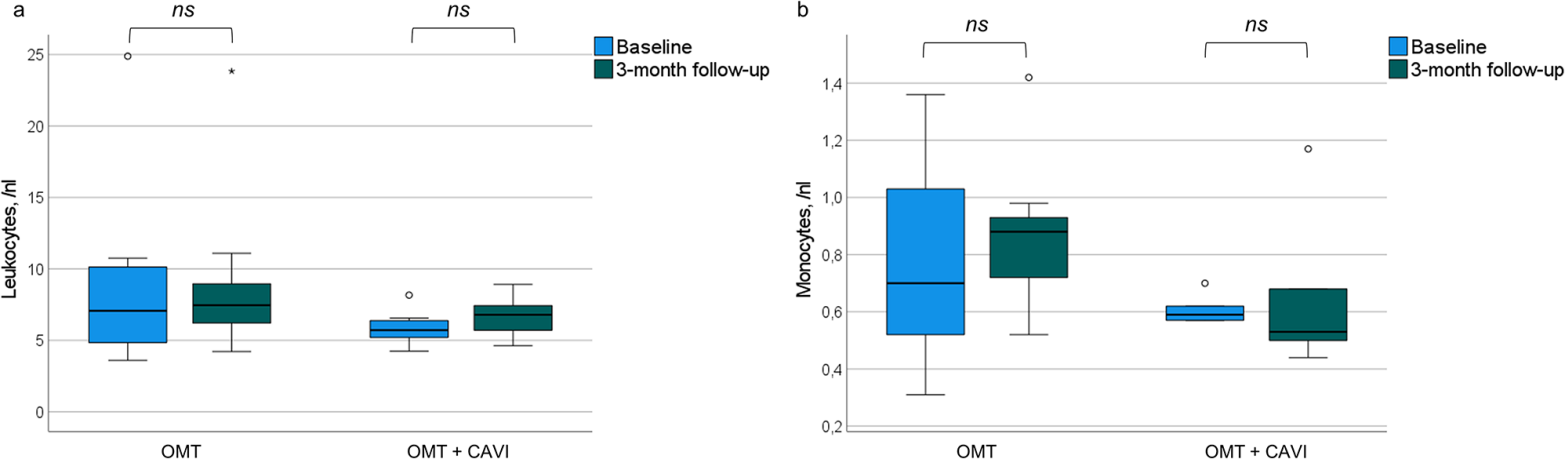
Circulating absolute leukocyte (a, OMT + CAVI *n* = 8, OMT *n* = 10) and monocyte (b, OMT + CAVI *n* = 7, OMT *n* = 10) counts in patients with OMT (optimal medical therapy) + CAVI (inferior caval valve implantation) or OMT at baseline and three-month follow-up; ns = not significant. ○ indicates the exceeding of the 1.5-fold interquartile range (IQR); * indicates the exceeding of the 3-fold IQR

**Fig. 2:**
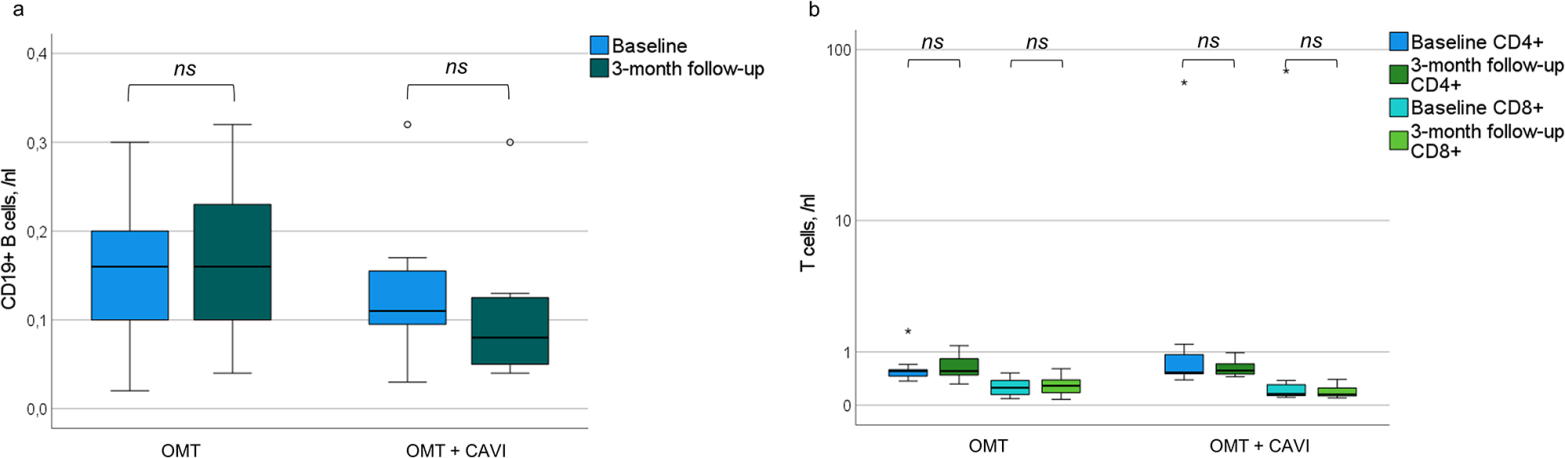
Absolute cell counts of CD19 + B cells (a, OMT + CAVI *n* = 7, OMT *n* = 9) and CD4 + T cells (b, logarithmic scale, OMT + CAVI *n* = 7, OMT *n* = 10) as well as CD8 + T cells (b, logarithmic scale due to outliers, OMT + CAVI *n* = 7, OMT *n* = 10) in patients with OMT (optimal medical therapy) + CAVI (inferior caval valve implantation) or OMT at baseline and three-month follow-up; ns = not significant. ○ indicates the exceeding of the 1.5-fold interquartile range (IQR); * indicates the exceeding of the 3-fold IQR

The intragroup comparison after three months did not reveal relevant differences in leukocyte, lymphocyte, neutrophil, eosinophil, basophil, and monocyte cell counts in the OMT + CAVI and OMT group compared to baseline (Fig. 1). In the OMT + CAVI group, we measured a significant difference in the relative distribution of leukocytes subpopulations with an increase in relative levels of CD8 + T cells (9.0 [6.0–27.0] vs. 12.0 [11.0–26.0] in percentage of lymphocytes, *p* < 0.05, *z* = -2.043) after three months compared to baseline. There were no differences in absolute counts of circulating CD8 + T cells between baseline and three-month follow-up (Fig. 2). Additionally, we observed a decrease of the proportion of CD19 + B cells in the OMT + CAVI group (10.0 [5.0–18.0], *n* = 7, vs. 6.0 [4.0–13.0], *n* = 7, in percentage of lymphocytes, *p* < 0.05, *z* = -2.047). Further trends after OMT + CAVI comprised the course of T cells and natural killer cells: The relative levels of CD4 + T cells increased, while relative levels of regulatory T cells decreased after OMT + CAVI. Moreover, we observed an increase in relative levels of natural killer cells between baseline and the three-month follow-up. None of these trends achieved statistical significance. Concerning other immune cell subpopulations, there were no significant intragroup differences between baseline and three-months follow up in the OMT + CAVI and OMT group (Table 3, supplemental material figures S1–S11).

**Table 3:** Circulating immune cells drawn from a peripheral vein at baseline and three-month follow-up

	OMT (*n* = 10)				OMT + CAVI (*n* = 8)			
	Baseline	3 months	*p* for intragroup comparison	*z* value for intragroup comparison	Baseline	3 months	*p* for intragroup comparison	*z* value for intragroup comparison
Leukocytes (IQR), /nl	7.1 (4.8–10.3)	7.5 (6.1–9.5)	0.799	-0.255	5.7 (5.1–6.5)	6.8 (5.4–7.5)	0.093	-1.680
Lymphocytes (IQR), /nl	1.1 (1.0-1.5) (*n =* 9)	1.4 (0.9–1.8) (*n =* 9)	0.594	-0.533	1.1 (0.8–1.3) (*n* = 5)	1.0 (1.0-1.1) (*n* = 5)	0.686	-0.405
Neutrophils (IQR), /nl	5.5 (3.0-7.7) (*n* = 8)	5.3 (3.8–7.7) (*n* = 8)	0.889	-0.140	3.7 (3.4–4.4) (*n* = 7)	4.8 (3.3–7.2) (*n* = 7)	0.237	-1.183
Eosinophils (IQR), /nl	0.2 (0.1–0.4) (*n* = 8)	0.2 (0.1–0.3) (*n* = 8)	0.261	-1.123	0.2 (0.1–0.4) (*n* = 5)	0.2 (0.1–0.4) (*n* = 5)	0.686	-0.405
Basophils (IQR), /nl	0.1 (0.0-0.1) (*n* = 9)	0.1 (0.1–0.1) (*n* = 9)	0.672	-0.423	0.1 (0.0-0.5) (*n* = 5)	0.0 (0.0-0.1) (*n* = 5)	0.593	-0.535
Monocytes (IQR), /nl	0.6 (0.4–0.8)	0.7 (0.6–0.8)	0.678	-0.415	0.5 (0.4–0.6) (*n* = 7)	0.4 (0.4–0.5) (*n* = 7)	0.176	-1.352
CD4 + CD8 + cell ratio (IQR)	2.7 (1.2–4.8)	2.4 (1.0-4.4)	0.553	-0.594	4.9 (1.6–7.3) (*n* = 7)	4.1 (1.7–5.7) (*n* = 7)	0.310	-1.014
CD8- CD4- T cells (IQR), percent of T cells	2.5 (0.9-4,9)	2.0 (1.1–4.3)	0.575	-0.561	3.5 (1.0-5.1) (*n* = 7)	3.7 (1.4–5.8) (*n* = 7)	0.237	-1.183
CD8 + CD4 + T cells (IQR), percent of T cells	1.2 (0.7–2.7)	1.2 (0.9–2.2)	0.906	-0.119	1.4 (1.2–1.7) (*n* = 7)	1.7 (1.1–2.1) (*n* = 7)	0.735	-0.338
CD3 + T cells (IQR), percent of lymphocytes	71.0 (65.0-78.5)	74.0 (70.0-80.5)	0.610	-0.510	65.0 (37.0–76.0) (*n* = 7)	72.0 (63.0–85.0) (*n* = 7)	0.128	-1.524
CD4 + T cells (IQR), percent of lymphocytes	44.5 (39.5–53.8)	46.5 (34.8–59.5)	0.905	-0.119	45.0 (28.0–51.0) (*n* = 7)	48.0 (45.0–64.0) (*n* = 7)	0.395	-0.851
CD4 + T cells (IQR), percent of T cells	68.0 (49.8–79.9)	68.2 (47.1–79.2)	0.575	-0.561	71.6 (53.9–83.1) (*n* = 7)	75.9 (58.1–80.4) (*n* = 7)	0.735	-0.338
CD8 + T cells (IQR), percent of lymphocytes	18.5 (11.8–34.0)	19.0 (13.8–34.3)	0.497	-0.680	9.0 (6.0–27.0) (*n* = 7)	12.0 (11.0- 26.0) (*n* = 7)	0.041	-2.043
CD8 + T cells (IQR), percent of T cells	26.2 (17.3–44.4)	29.3 (18.1–44.7)	0.508	-0.663	15.5 (14.0-35.9) (*n* = 7)	18.7 (14.5–35.0) (*n* = 7)	0.612	-0.507
CD28 + T cells (IQR), percent of CD8- T cells	92.5 (85.0-97.5)	92.5 (86.8–98.3)	0.404	-0.834	97.0 (88.5–99.0) (*n* = 6)	95.5 (87.6–99.0) (*n* = 6)	0.180	-1.342
CD28 + T cells (IQR), percent of CD8 + T cells	38.5 (22.0-53.5)	34.0 (26.5–55.8)	0.646	-0.459	62.0 (27.8–68.5) (*n* = 6)	59.5 (30.8–67.8) (*n* = 6)	0.916	-0.105
CD57 + T cells (IQR), percent of CD8- T cells	7.0 (3.3–13.5)	7.5 (2.5–13.3)	0.670	-0.426	4.0 (1.0-10.3) (*n* = 6)	6.0 (2.0–10.0) (*n* = 6)	0.129	-1.518
CD57 + T cells (IQR), percent of CD8 + T cells	57.5 (43.8–71.5)	64.5 (36.0-69.3)	0.959	-0.051	35.5 (23.5–49.3) (*n* = 6)	35.0 (26.8–51.0) (*n* = 6)	0.458	-0.742
HLA-DR + T cells (IQR), percent of CD8- T cells	12.5 (6.0-18.5)	11.0 (7.0-15.3)	0.799	-0.255	9.0 (5.5–13.0) (*n* = 6)	9.5 (8.5–17.8) (*n* = 6)	0.498	-0.677
HLA-DR + T cells (IQR), percent of CD8 + T cells	31.0 (11.8–43.3)	25.0 (15.5–48.5)	0.953	-0.059	18.5 (11.0–47.0) (*n* = 6)	27.5 (20.0-37.5) (*n* = 6)	0.600	-0.524
CD3 + CD8 + cells (IQR), percent of CD3 + cells	22.6 (16.2–44.5) (*n* = 9)	31.1 (18.6–44.5) (*n* = 9)	0.260	-1.125	20.2 (13.6–37.1) (*n* = 5)	23.6 (13.2–30.6) (*n* = 5)	0.225	-1.214
CD3 + CD8- cells (IQR), percent of CD3 + cells	72.3 (48.6–81.0) (*n* = 9)	66.6 (47.4–79.7) (*n* = 9)	0.374	-0.889	72.2 (56.6–80.9) (*n* = 5)	66.8 (62.0-81.6) (*n* = 5)	0.345	-0.944
naive CD45RA + CCR7 + cells (IQR), percent of CD8 + cells	5.5 (4.2–10.7) (*n* = 9)	5.9 (4.2–12.1) (*n* = 9)	0.441	-0.770	3.4 (3.1–10.9) (*n* = 5)	4.3 (2.4–14.4) (*n* = 5)	0.893	-0.135
TEMRA CD45RA + CCR7- cells (IQR), percent of CD8 + cells	45.8 (34.0-64.2) (*n* = 9)	51.6 (27.3–63.0) (*n* = 9)	0.214	-1.244	33.4 (23.1–59.0) (*n* = 5)	26.9 (23.2–57.7) (*n* = 5)	0.893	-0.135
Naive CD45 + CCR7 + cells (IQR), percent of CD4 + cells	27.6 (14.0-36.2) (*n* = 9)	23.9 (15.3–39.8) (*n* = 9)	0.374	-0.889	19.2 (10.0-21.6) (*n* = 5)	23.1 (7.6–25.1) (*n* = 5)	0.500	-0.674
TEMRA CD45RA + CCR7- cells (IQR), percent of CD4 + cells	0.8 (0.1-4.0) (*n* = 9)	0.2 (0.2–1.2) (*n* = 9)	0.260	-1.126	1.0 (0.1–2.5) (*n* = 5)	1.5 (0.4–2.1) (*n* = 5)	0.893	-0.135
CD45RA + cells (IQR), percent of CD4 + cells	28.8 (16.2–36.6) (*n* = 9)	24.0 (16.5–40.0) (*n* = 9)	0.515	-0.652	20.4 (10.5- 23.5) (*n* = 5)	25.2 (8.9–26.1) (*n* = 5)	0.225	-1.214
CD45RA- cells (IQR), percent of CD4 + cells	71.2 (63.4–83.8) (*n* = 9)	76.0 (60.0-83.5) (*n* = 9)	0.374	-0.889	79.6 (76.5–89.5) (*n* = 5)	74.9 (73.9–91.1) (*n* = 5)	0.225	-1.214
CD25 + CD127- T regulatory cells (IQR), percent of CD4 + cells	6.9 (5.8–9.1) (*n* = 9)	6.5 (5.9–7.3) (*n* = 9)	0.214	-1.244	9.5 (8.8–9.8) (*n* = 5)	8.3 (6.3–9.1) (*n* = 5)	0.138	-1.483
CD19 + B cells (IQR), percent of lymphocytes	9.5 (7.0-13.3)	12.0 (7.5–15.0)	0.095	-1.672	10.0 (5.0–18.0) (*n* = 7)	6.0 (4.0–13.0) (*n* = 7)	0.041	-2.047
Natural killer cells (IQR), percent of lymphocytes	14.5 (10.8–16.3)	13.5 (11.3–19.5)	0.527	-0.632	14.0 (10.0–19.0) (*n* = 7)	17.0 (11.0–24.0) (*n* = 7)	0.499	-0.676

Continuous variables are shown as median and interquartile ranges (IQR) due to the distribution of parameters (uniform per variable). OMT, optimal medical therapy; CAVI, caval valve implantation; *n*, number of patients with laboratory measurements in case of missing data

### Circulating inflammatory mediators

**Fig. 3:**
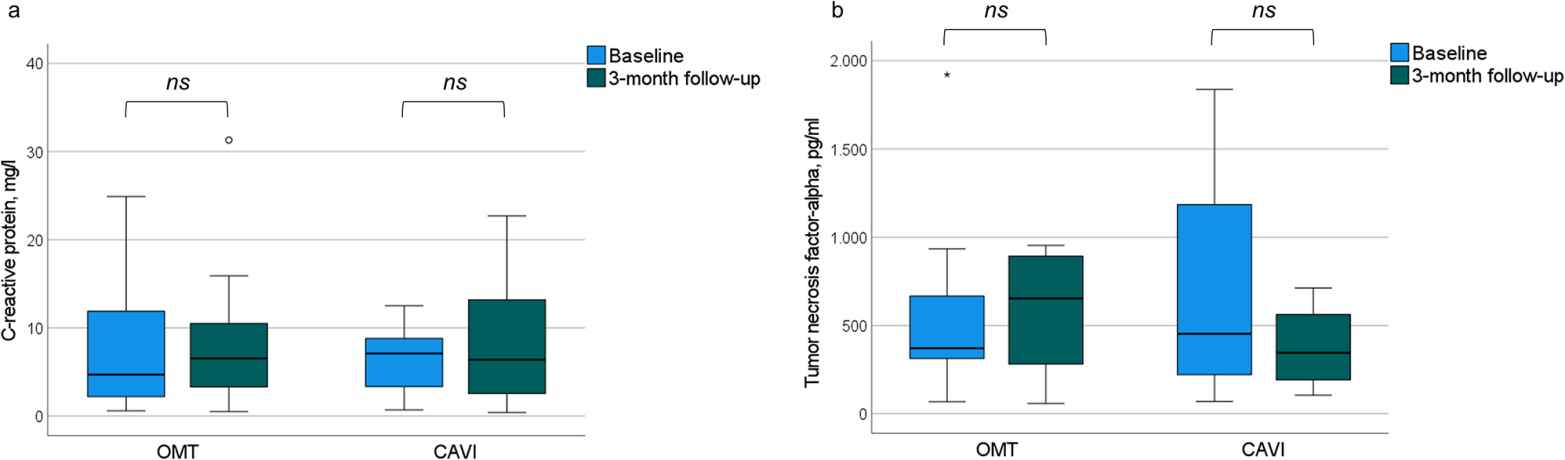
Values of C-reactive protein (a, OMT + CAVI *n* = 8, OMT *n* = 10) and tumor necrosis factor-alpha (b, OMT + CAVI *n* = 5, OMT *n* = 7) in patients with OMT (optimal medical therapy) + CAVI (inferior caval valve implantation) or OMT at baseline and three-month follow-up; ns = not significant. ○ indicates the exceeding of the 1.5-fold interquartile range (IQR). * indicates the exceeding of the 3-fold IQR

Levels of circulating TNF-α slightly increased between baseline and three-month follow up in the OMT + CAVI group; however, the changes in levels of TNF-α and CRP did not differ significantly between baseline and three-month follow-up in the OMT + CAVI or OMT group. Levels of concanavalin A-induced lymphocytic IFN-γ, IL-2, -4, -5, -10-, and four hours lipopolysaccharides stimulated TNF-α did not change between baseline and three-month follow-up in the OMT + CAVI or OMT group (Table 4; Fig. 3, supplemental material figures S12-S19).

**Table 4:** Circulating inflammatory markers drawn from a peripheral vein at baseline and three-month follow-up

	OMT (*n* = 10)				OMT + CAVI (*n* = 8)			
	Baseline	3 months	*p* for intragroup comparison	*z* value for intragroup comparison	Baseline	3 months	*p* for intragroup comparison	*z* value for intragroup comparison
Non-stimulated								
C-reactive protein (IQR), mg/l	4.7 (2.1–12.8)	6.6 (2.9–11.9)	0.959	-0.051	7.1 (2.6–8.9)	6.4 (2.4–16.1)	0.889	-0.140
Tumor necrosis factor-alpha (IQR), pg/ml	16.0 (8.6–29.1) (*n* = 7)	18.1 (14.0-30.1) (*n* = 7)	0.672	-0.423	13.8 (9.4–15.8) (*n* = 5)	19.1 (11.3–31.1) (*n* = 5)	0.080	-1.753
Stimulated								
Stimulated tumor necrosis factor-alpha (4 h LPS) (IQR), pg/ml	1275.0 (1020.0-2583.0)	1140.0 (15.7-2239.8)	0.386	-0.866	1180.0 (869.0-1353.0) (*n* = 5)	909.0 (286.4–1523.0) (*n* = 5)	0.345	-0.944
Interferon-gamma (IQR), pg/ml (ConA)	924.0 (523.0-1350.0) (*n* = 7)	1093.0 (626.0- 2969.0) (*n* = 7)	1.000	0.000	1279.5 (460.0-3095.8) (*n* = 4)	951.0 (412.0-1467.5) (*n* = 4)	0.273	-1.095
Interleukin-2 (IQR), pg/ml (ConA)	578.0 (283.0-854.0) (*n* = 7)	660.0 (343.0-1024.0) (*n* = 7)	0.310	-1.014	814.5 (530.3–1137.0) (*n* = 4)	585.5 (434.0- 641.0) (*n* = 4)	0.144	-1.461
Interleukin-4 (IQR), pg/ml (ConA)	13.0 (8.0–27.0) (*n* = 7)	11.0 (9.0–24.0) (*n* = 7)	0.674	-0.420	13.5 (11.5–18.5) (*n* = 4)	17.5 (11.5–19.0) (*n* = 4)	0.461	-0.736
Interleukin-5 (IQR), pg/ml (ConA)	10.0 (0.0–18.0) (*n* = 7)	9.0 (1.0–21.0) (*n* = 7)	0.933	-0.085	7.5 (1.3–25.8) (*n* = 4)	20.0 (6.0–34.0) (*n* = 4)	0.273	-1.095
Interleukin-10 (IQR), pg/ml (ConA)	37.0 (25.0–66.0) (*n* = 7)	41.0 (13.0–56.0) (*n* = 7)	0.398	-0.845	26.0 (20.3–50.5) (*n* = 4)	31.5 (27.0-38.3) (*n* = 4)	0.713	-0.368

Continuous variables are shown as median and interquartile ranges (IQR) due to the distribution of parameters (uniform per variable). OMT, optimal medical therapy; CAVI, caval valve implantation; *n*, number of patients with laboratory measurements in case of missing data. LPS, Lipopolysaccharides; Con A, concanavalin A-induced lymphocytic IFN-γ, TNF-α, IL-2, IL-4, IL-5, and IL-10 secretion

## Discussion

To the best of our knowledge, the present subanalysis of TRICAVAL is the first study evaluating the effect of OMT + CAVI on circulating immune cells and inflammatory markers in patients with severe TR. We initially hypothesized that the reduction of abdominal congestion following OMT + CAVI leads to an attenuation of systemic inflammation with a shift to a more anti-inflammatory pattern of circulating inflammatory markers. In the OMT + CAVI group, reduction of abdominal-venous congestion was confirmed by echocardiographic measurements at three-month follow-up [16]. This was not followed by substantial changes in levels of circulating immune cell subpopulations, levels of circulating inflammatory mediators (CRP and TNF-α), or the excretion of inflammatory mediators from stimulated lymphocytes in both groups – OMT + CAVI and OMT. Inflammatory mediator TNF-alpha and anti-inflammatory mediator IL-4 showed both a slight increase, whereas other inflammatory (CRP) and anti-inflammatory (IL-10) markers remained stable; thus, not indicating a clear tendency toward a more inflammatory or anti-inflammatory pattern after the procedure [17, 18]. In the OMT + CAVI group, the observed increase of the proportion of CD8 + T cells of the total amount of lymphocytes (but not of the total amount of T cells) and the decrease in the proportion of CD19 + B cells at three-month follow-up reached statistical significance. Additional trends comprised an increase in CD4 + T cells, natural killer cells and a decrease in the proportion of regulatory T cells. The observed changes in CD8 + and CD4 + T cells may indicate an enhancement of immune system activity, while the decrease in CD19 + B cells was reported to be accompanied by reduced immunoglobulins and an increased risk of infection [19–23]. In the present hypothesis-generating study, the observed changes in the distribution of immune cells do not point to a clear inflammatory or anti-inflammatory pattern. A subanalysis of patients with optimal CAVI excluding one patient with paravalvular leakage showed no further relevant differences regarding circulating immune cells and inflammatory mediators compared to OMT + CAVI patients (tables S1 and S2). Therefore, due to the lack of a consistent pattern in changes of immune cell subpopulations in the background of the small sample size and broad variations within each parameter, the observed changes may not be related to OMT + CAVI and are interpreted as clinically not relevant. In addition, there was no increase of severe infections in our patients at three-month follow-up indicated significant changes in the immune system.

Previous studies suggested that venous congestion results in an inflammatory response [4–7], which is also induced by an increase in oxidative stress due to congestion. In patients with cardiorenal syndrome, it was suggested that venous congestion enhances oxidative stress-mediated inflammation as shown by an increased infiltration of macrophages into the renal tissue [24]. Since OMT + CAVI leads to a relevant reduction of abdominal venous congestion, we expected a consistent anti-inflammatory response and/or a decrease of inflammatory markers after valve implantation. However, as OMT + CAVI does not treat TR itself, volume overload of the right ventricle persists. The response to ventricular volume overload involves the expression of inflammatory mediators in right ventricular tissue as shown in pulmonary hypertension in animal studies [25]. To our knowledge, there has been no confirmation by peripheral blood measurements in human studies so far. Nevertheless, it can be speculated that both pathways – on the one hand, a decrease in inflammation due to reduced abdominal venous congestion and, on the other hand, an inflammatory response due to extensive volume overload of the right ventricle - may have led to the divergent results in our study.

## Limitations

There are several limitations to the present subanalysis. The results of the TRICAVAL study are limited by the small number of study participants. After two valve dislocations and two stent migrations, the TRICAVAL study was stopped prematurely for safety concerns [11, 12]. The small sample size clearly limits the interpretation of the present findings; in particular as our patient population suffered from multiple comorbidities and the evaluated parameters showed broad variations in these patients. In addition, Inflammatory markers are prone to confounder such as medication (e.g. statins), environments, and clinical or subclinical infections [26, 27]. Thus, our results are regarded as hypothesis generating and should trigger subsequent larger, adequately powered studies on the effect of interventional TR approaches on inflammation. In the present study, we solely evaluated systemic effects in the blood compartment and changes in microenvironments such as the gastrointestinal tract, which is primarily affected by the reduction in congestion may have been missed. Therefore, the design of these subsequent studies should comprise analyses within different microenvironments including histopathological examinations of biopsies and the evaluation of oxidative stress parameters. Furthermore, it cannot be completely ruled out that the OMT + CAVI procedure itself causes an inflammatory response that can be detected up to three months after the procedure. Data from thoracoscopic TR surgery showed an increase in leukocytes and CRP on days three and four after surgery with a nearly normalized value of leukocytes and a slightly elevated CRP level at post-operative day six [28]. This does not point to a persisting inflammatory state after cardiac interventions in general.

Over the last years, new interventional therapies have been developed to treat severe TR. CAVI was one of the first interventional approaches used but was associated with periprocedural complications [11]. New dedicated heterotopic valve implantations such as TricValve (Products + Features GmbH, Vienna, Austria) and TRICENTO (Medira AG, Balingen, Germany) have a higher procedural and technical success rate [29, 30]. Both devices are implanted into the IVC and superior vena cava (SVC) and thus, addressing lower and upper venous congestion, which may result in a different inflammatory response in contrast to OMT + CAVI. In addition to the reduction of venous congestion, edge-to-edge techniques, annuloplasty systems, and orthotopic valve implantations lead to an additional decrease in right ventricular volume overload due to the treatment of the TR itself [31–34]. Beyond the reduction of venous congestion, these approaches may additionally lead to reduced expression of inflammatory mediators within the right heart. Further studies are needed to investigate the impact of new interventional TR therapies on inflammation and oxidative stress.

## Conclusion

The results of the present study suggest that reduction of venous congestion following OMT + CAVI may not result in clinically relevant changes in systemic inflammatory markers within a short-term follow-up.

### Electronic supplementary material

Below is the link to the electronic supplementary material.

Supplementary Material 1

Supplementary Material 2

Supplementary Material 3

Supplementary Material 4

Supplementary Material 5

Supplementary Material 6

Supplementary Material 7

Supplementary Material 8

Supplementary Material 9

Supplementary Material 10

Supplementary Material 11

Supplementary Material 12

Supplementary Material 13

Supplementary Material 14

Supplementary Material 15

Supplementary Material 16

Supplementary Material 17

Supplementary Material 18

Supplementary Material 19

Supplementary Material 20

## Data Availability

The data that support the findings of this study are available from the corresponding author upon reasonable request.

## Abbreviations

CAVI: Interventional valve implantation into the inferior vena cava
Con A: Concanavalin A
CRP: C-reactive protein
IFN-γ: Interferon-gamma
IL: Interleukin
IVC: Inferior vena cava
OMT: Optimal medical therapy
TNF-α: Tumor necrosis factor-alpha
TR: Tricuspid regurgitation
TRICAVAL: Treatment of Severe Secondary Tricuspid Regurgitation in Patients with Advance Heart Failure with Caval Vein Implantation of the Edwards Sapien XT Valve
SVC: Superior vena cava
