# Supplementary figures and images for: Effect of inferior caval valve implantation on circulating immune cells and inflammatory mediators in severe tricuspid regurgitation
Source: BMC Cardiovasc Disord. 2024 Jul 18;24:373. doi: 10.1186/s12872-024-04044-1 (PMC11256587; doi:10.1186/s12872-024-04044-1)

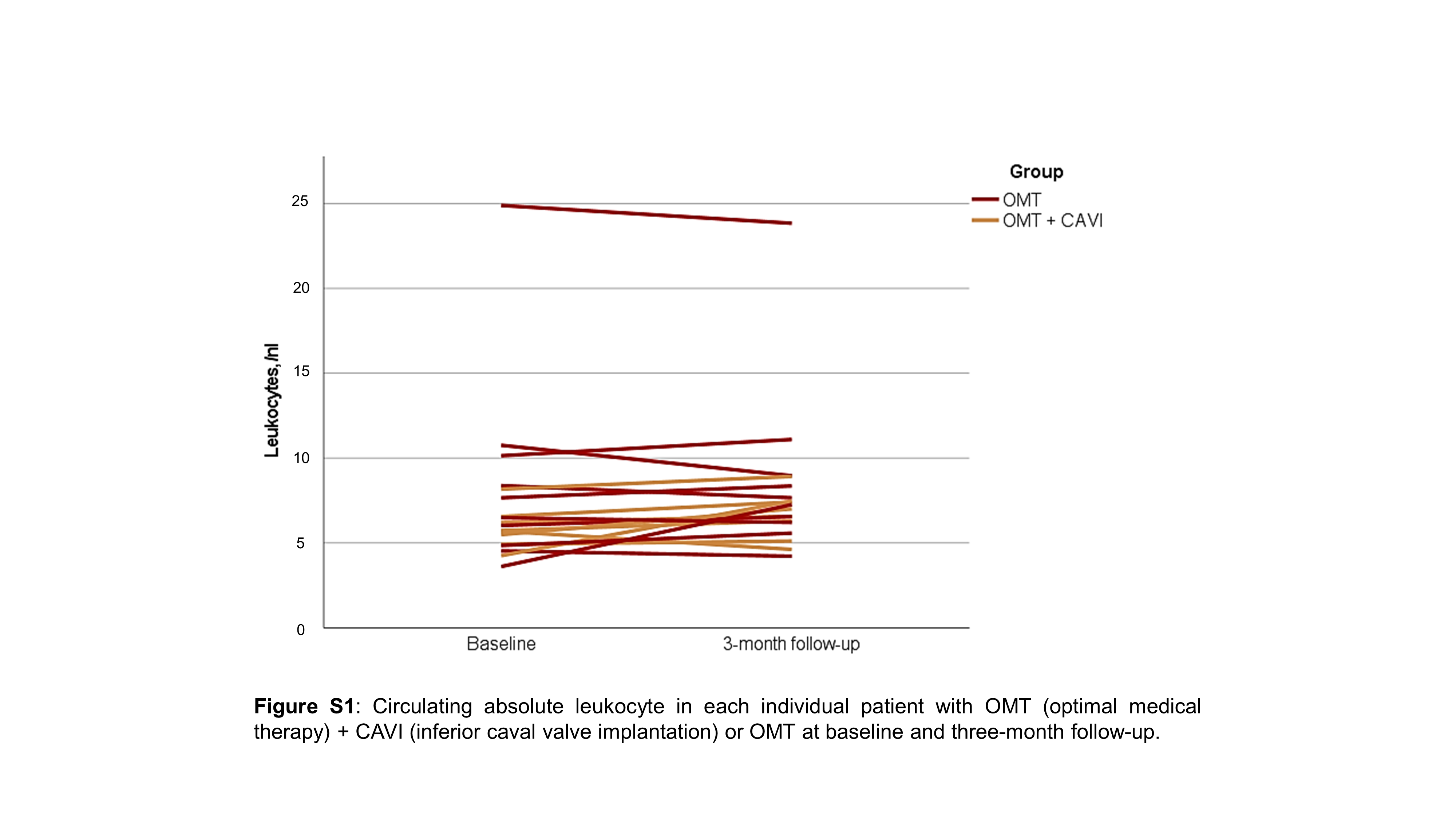

Supplement: Supplementary file 1 — Supplementary Material 1 [file 12872_2024_4044_MOESM1_ESM.tif]

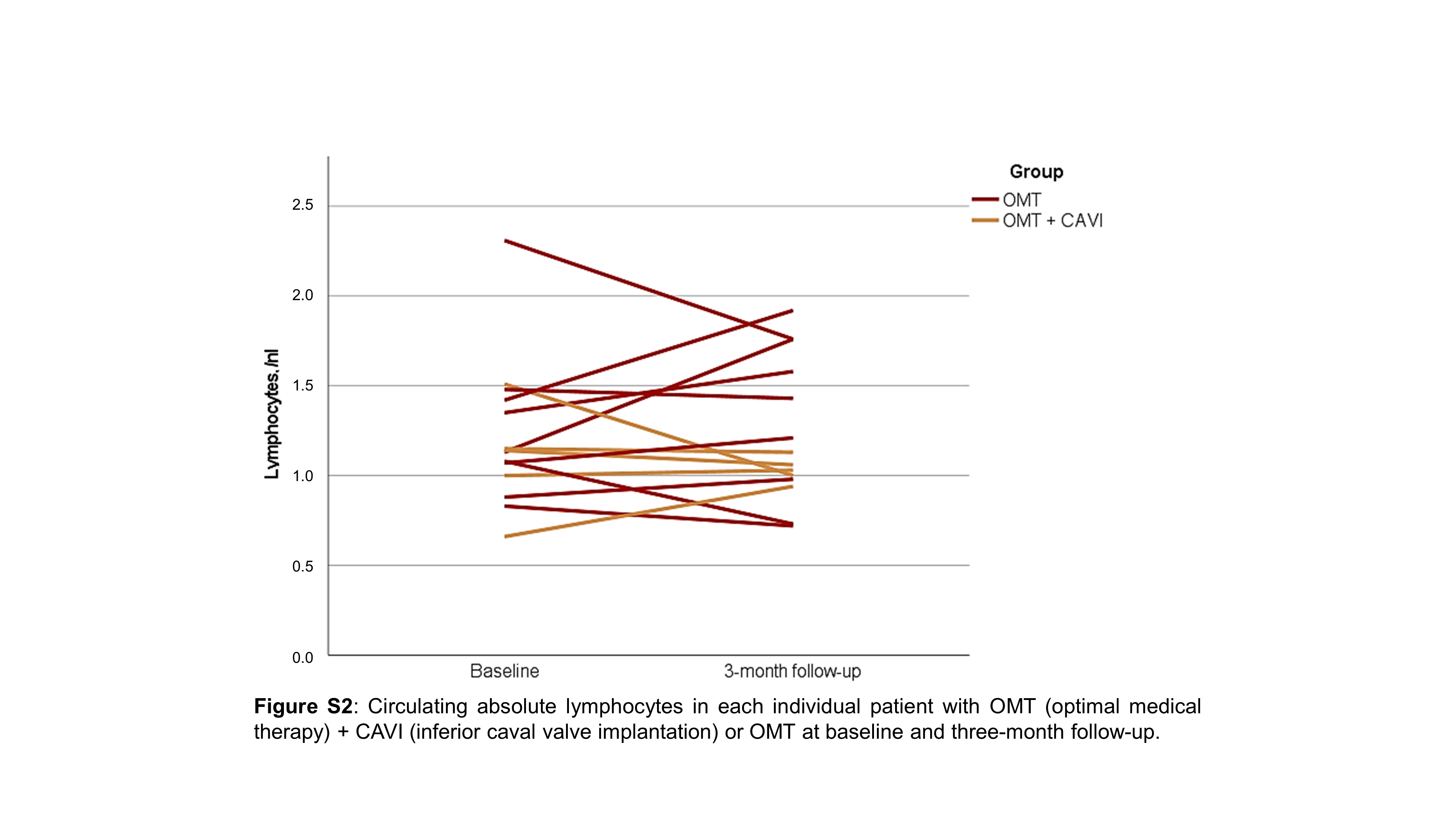

Supplement: Supplementary file 2 — Supplementary Material 2 [file 12872_2024_4044_MOESM2_ESM.tif]

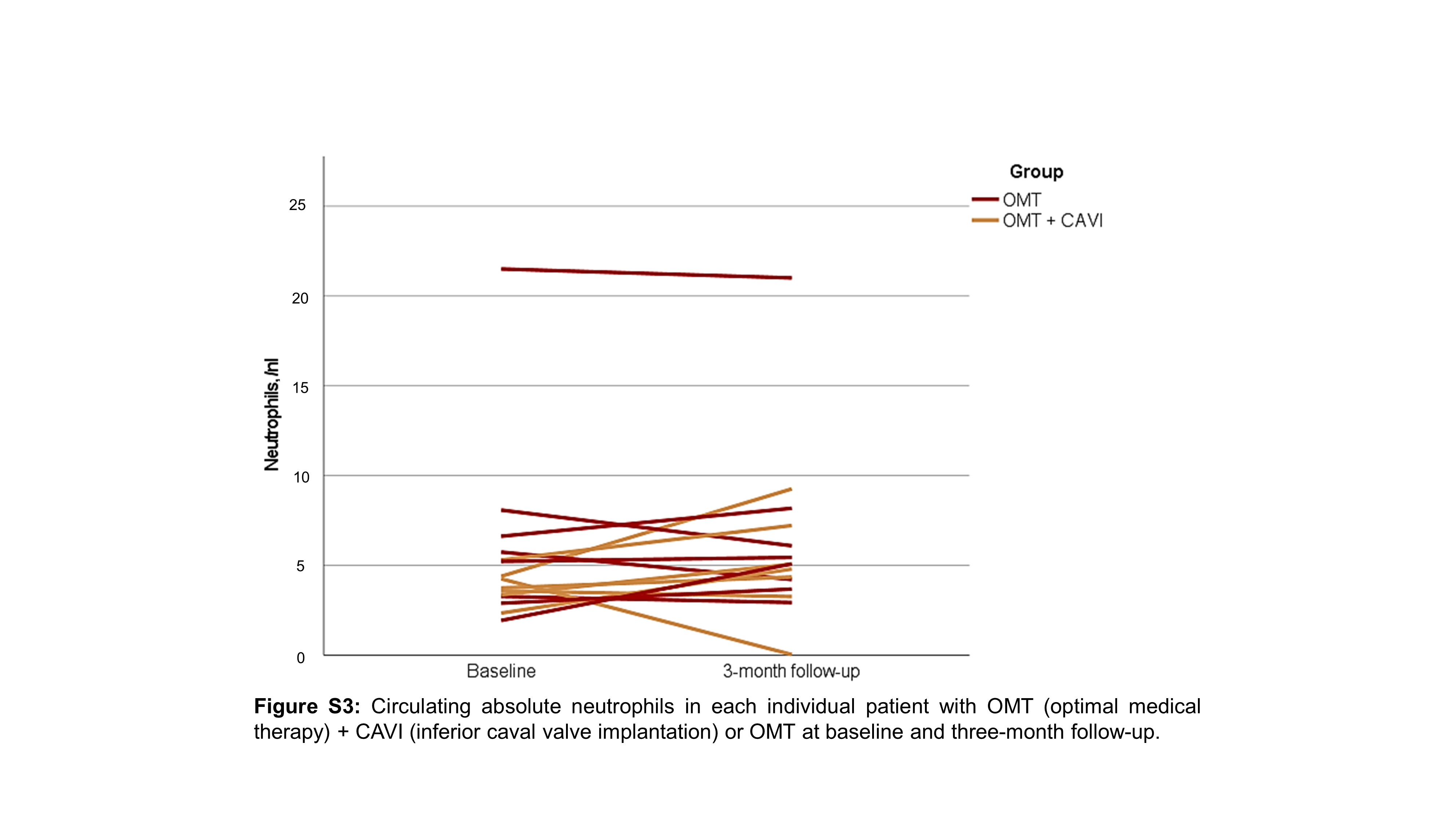

Supplement: Supplementary file 3 — Supplementary Material 3 [file 12872_2024_4044_MOESM3_ESM.tif]

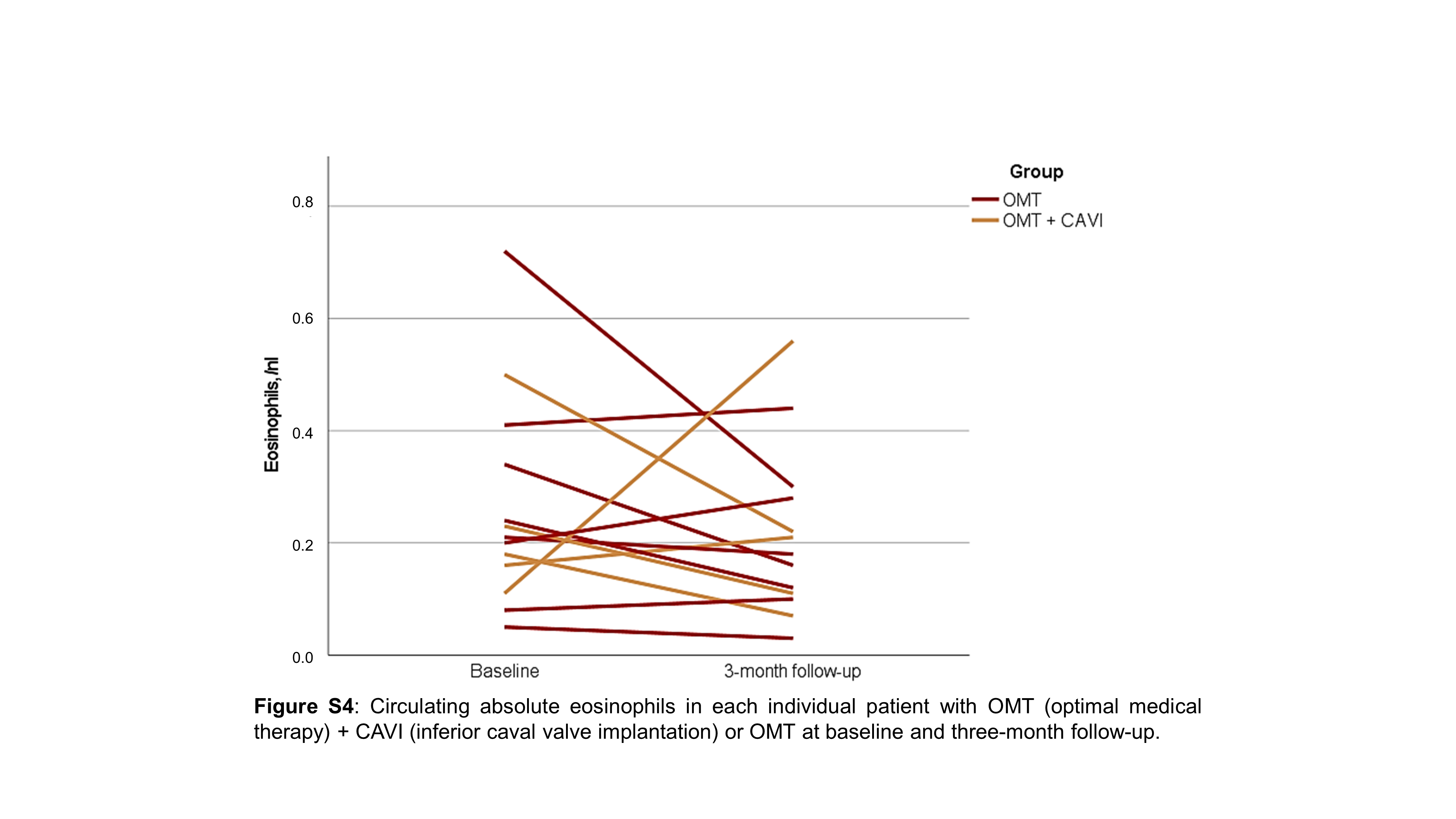

Supplement: Supplementary file 4 — Supplementary Material 4 [file 12872_2024_4044_MOESM4_ESM.tif]

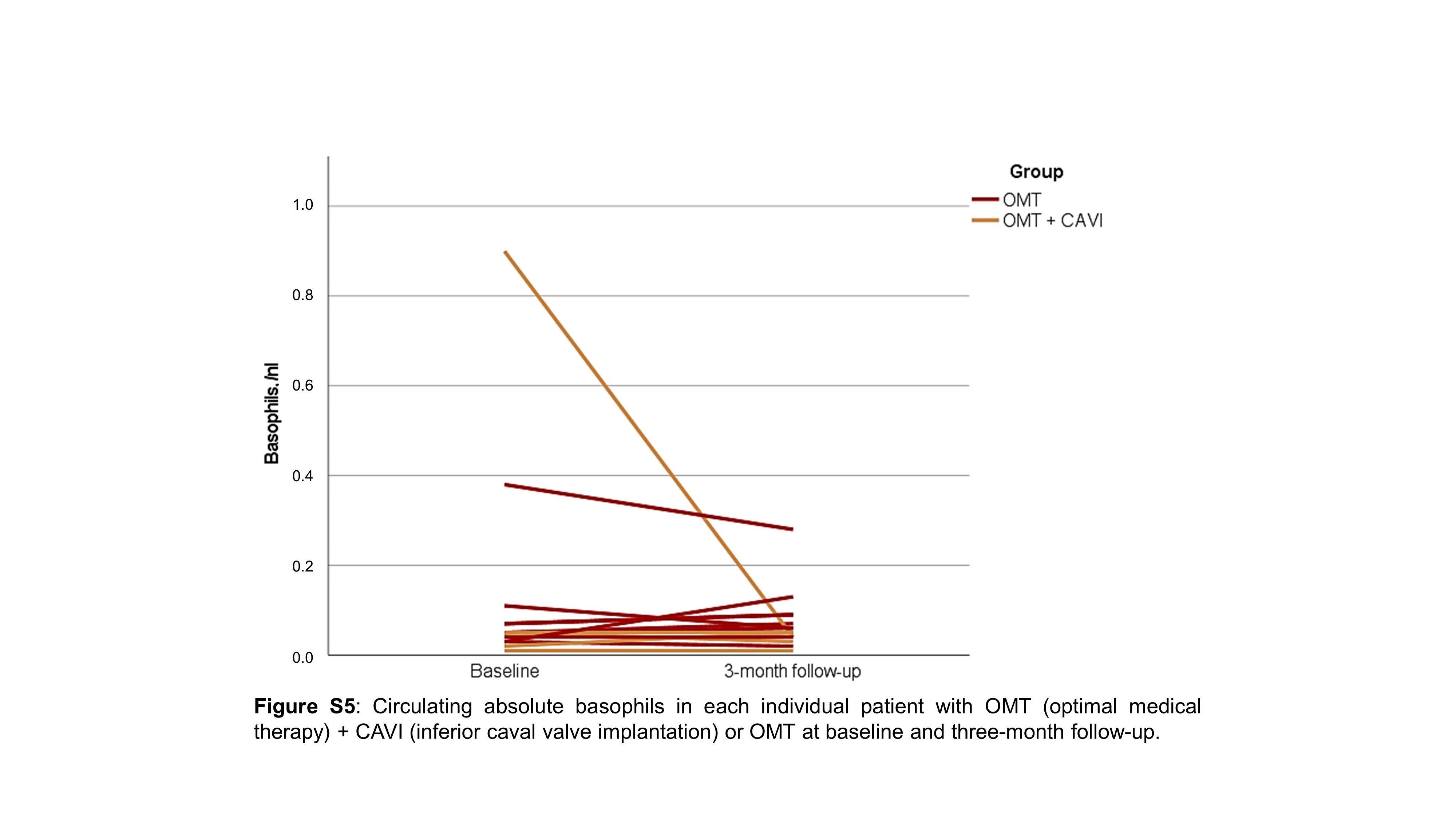

Supplement: Supplementary file 5 — Supplementary Material 5 [file 12872_2024_4044_MOESM5_ESM.tif]

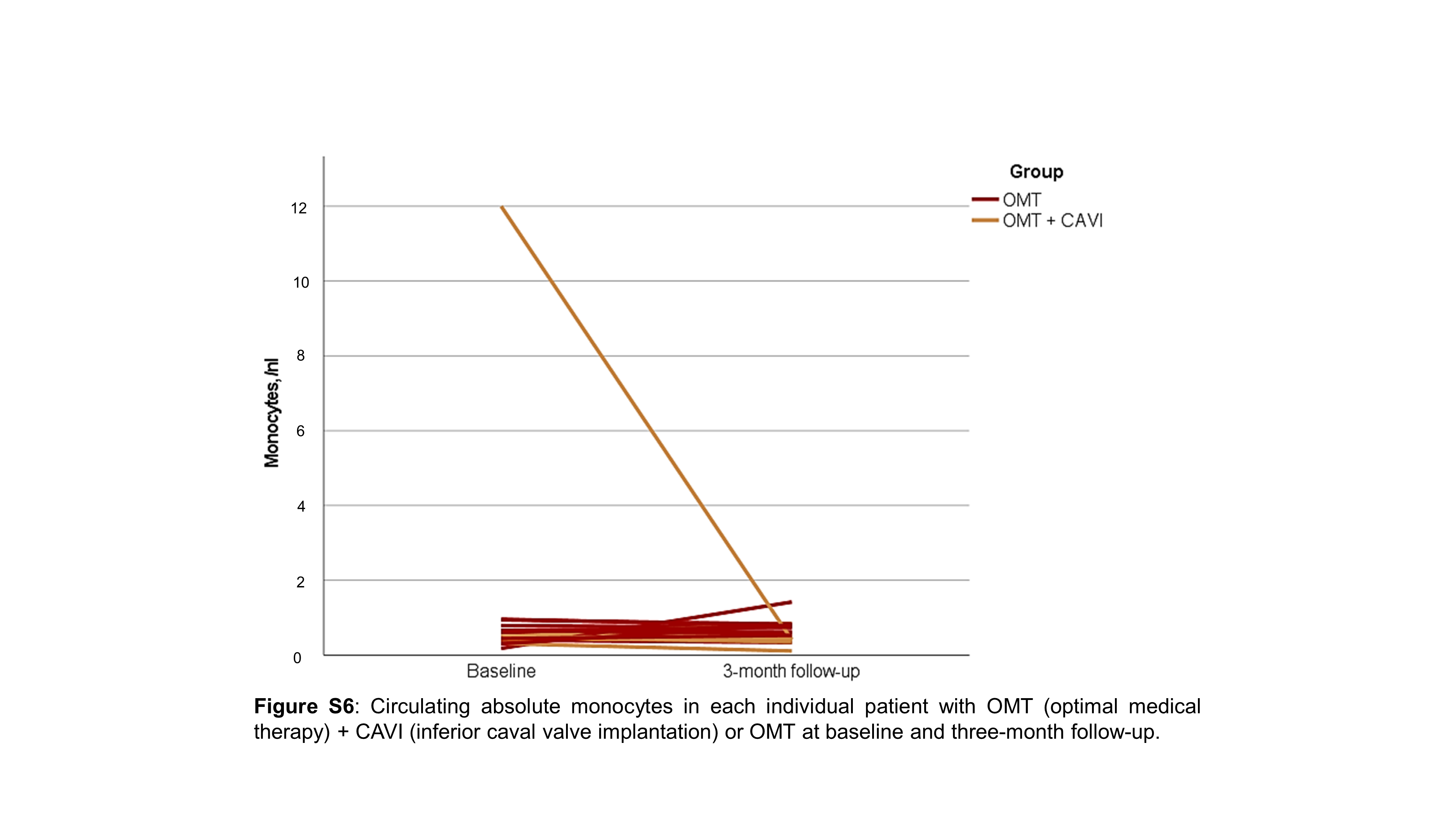

Supplement: Supplementary file 6 — Supplementary Material 6 [file 12872_2024_4044_MOESM6_ESM.tif]

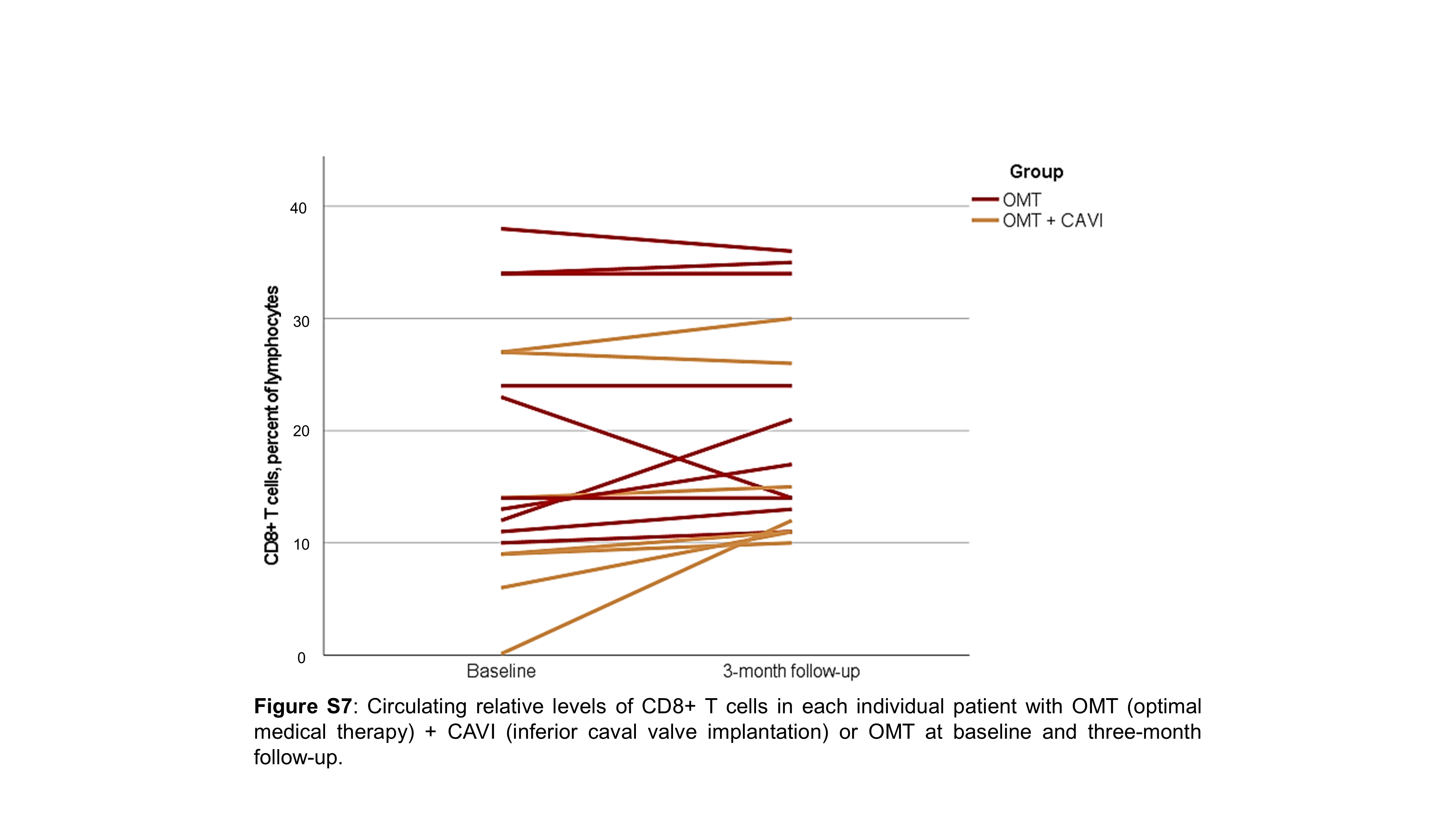

Supplement: Supplementary file 7 — Supplementary Material 7 [file 12872_2024_4044_MOESM7_ESM.tif]

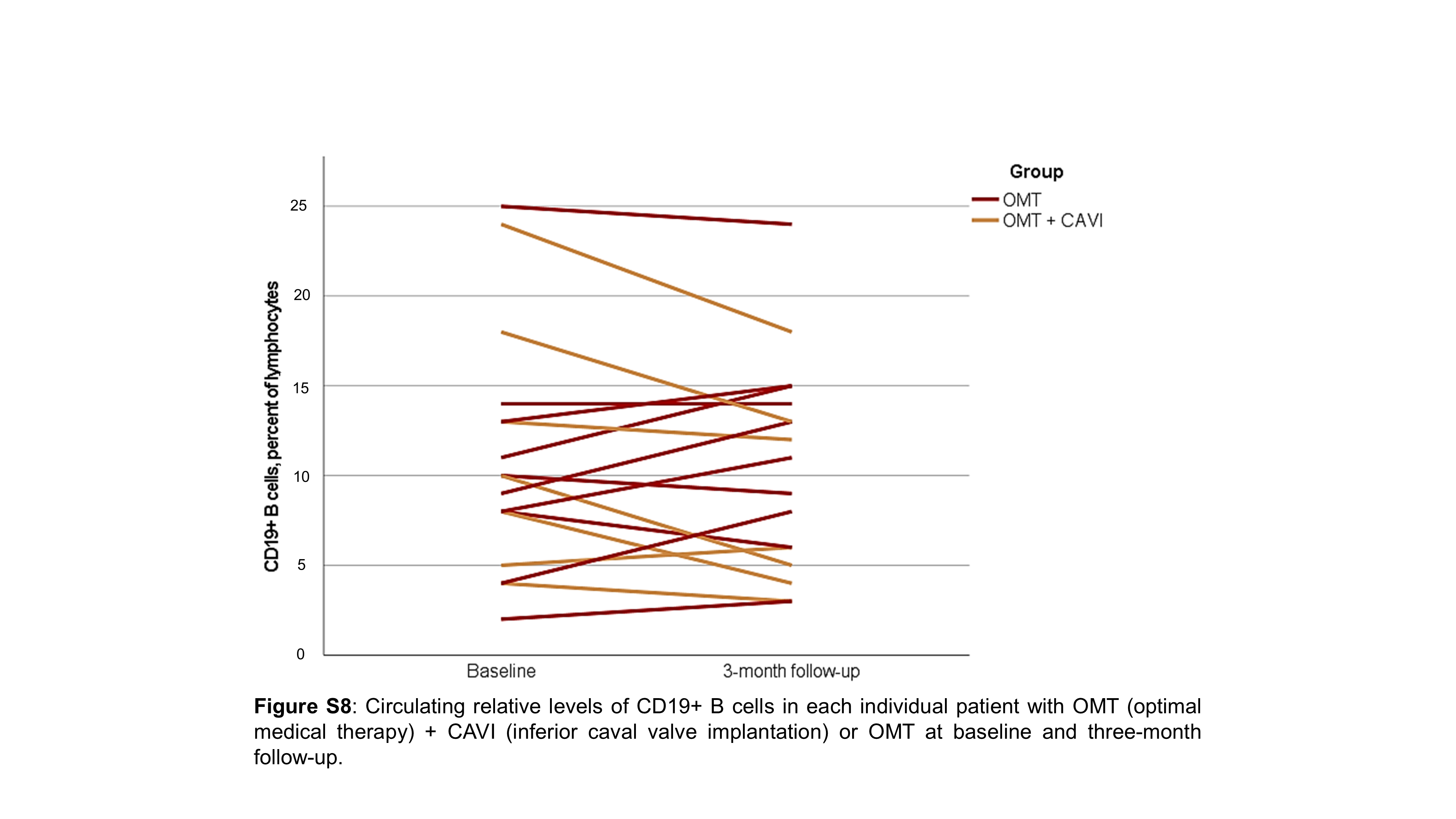

Supplement: Supplementary file 8 — Supplementary Material 8 [file 12872_2024_4044_MOESM8_ESM.tif]

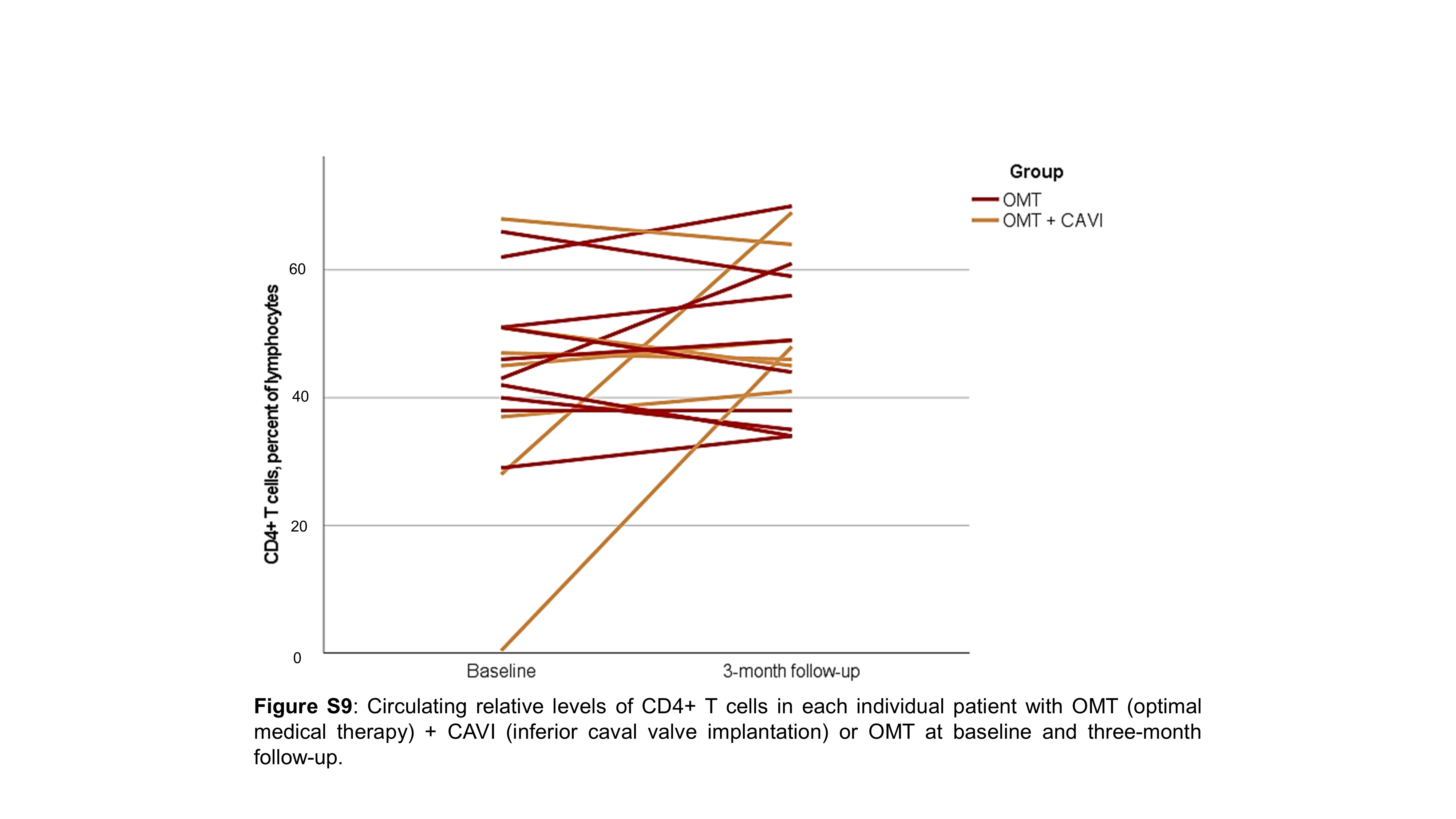

Supplement: Supplementary file 9 — Supplementary Material 9 [file 12872_2024_4044_MOESM9_ESM.tif]

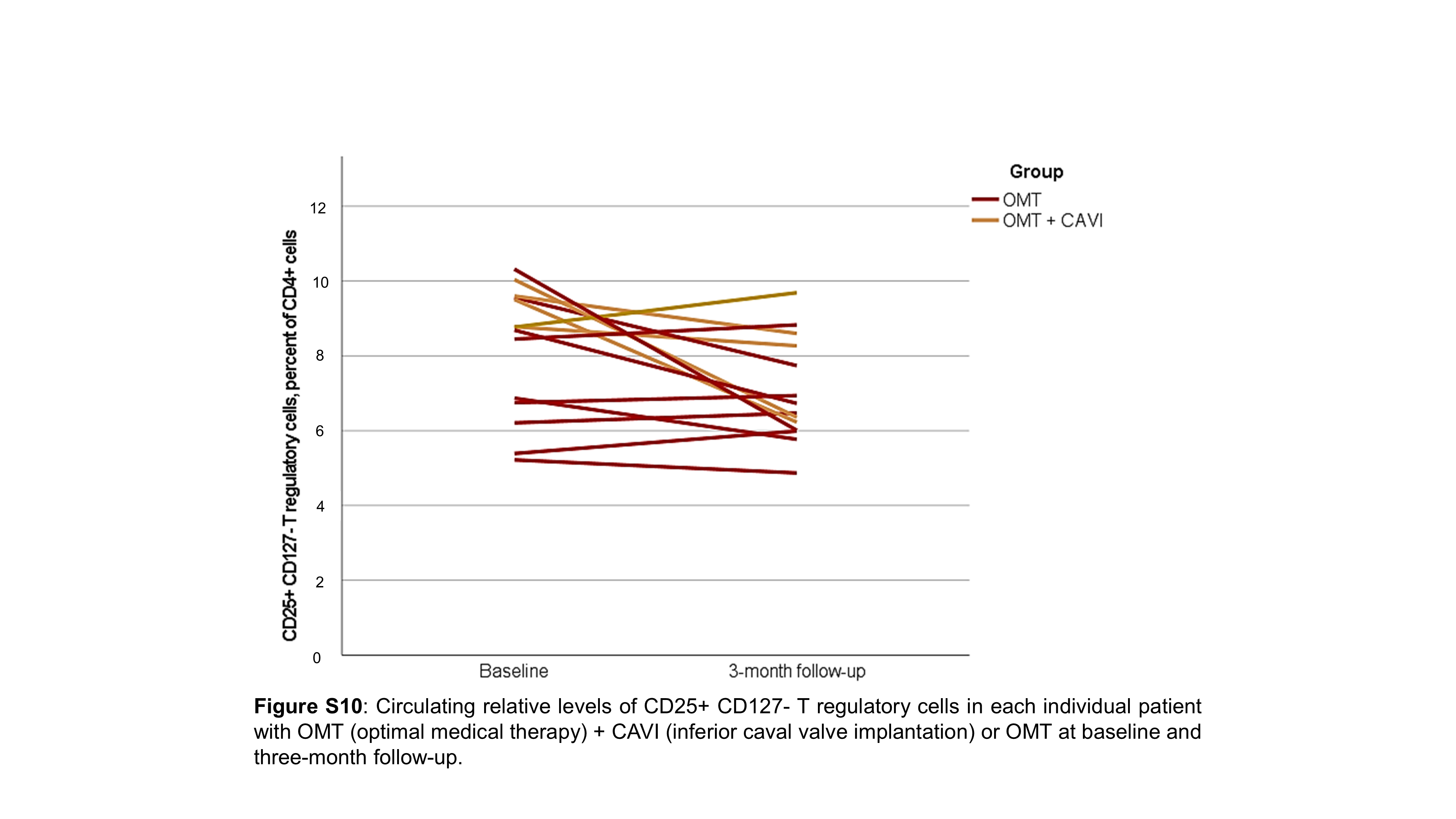

Supplement: Supplementary file 10 — Supplementary Material 10 [file 12872_2024_4044_MOESM10_ESM.tif]

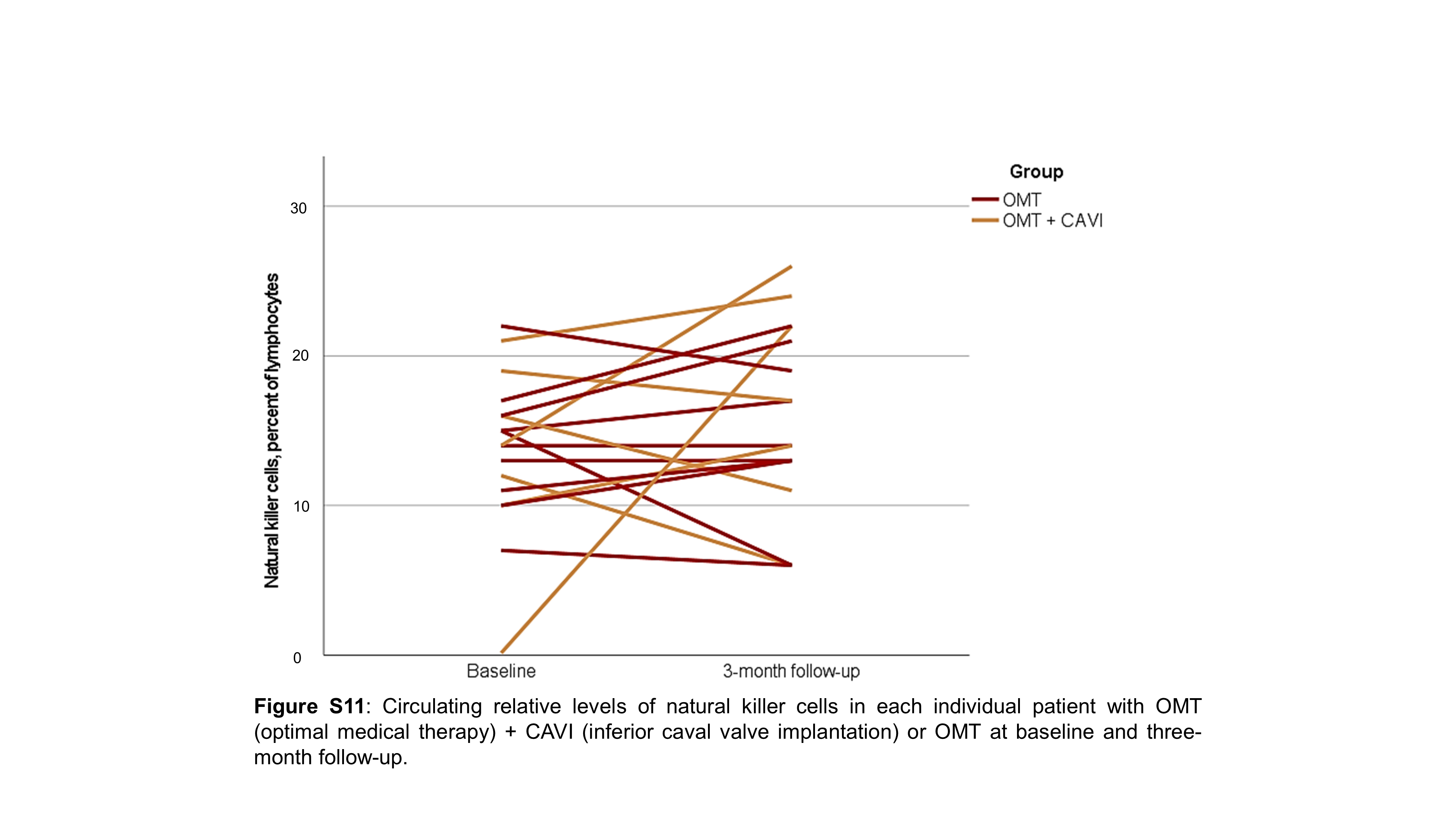

Supplement: Supplementary file 11 — Supplementary Material 11 [file 12872_2024_4044_MOESM11_ESM.tif]

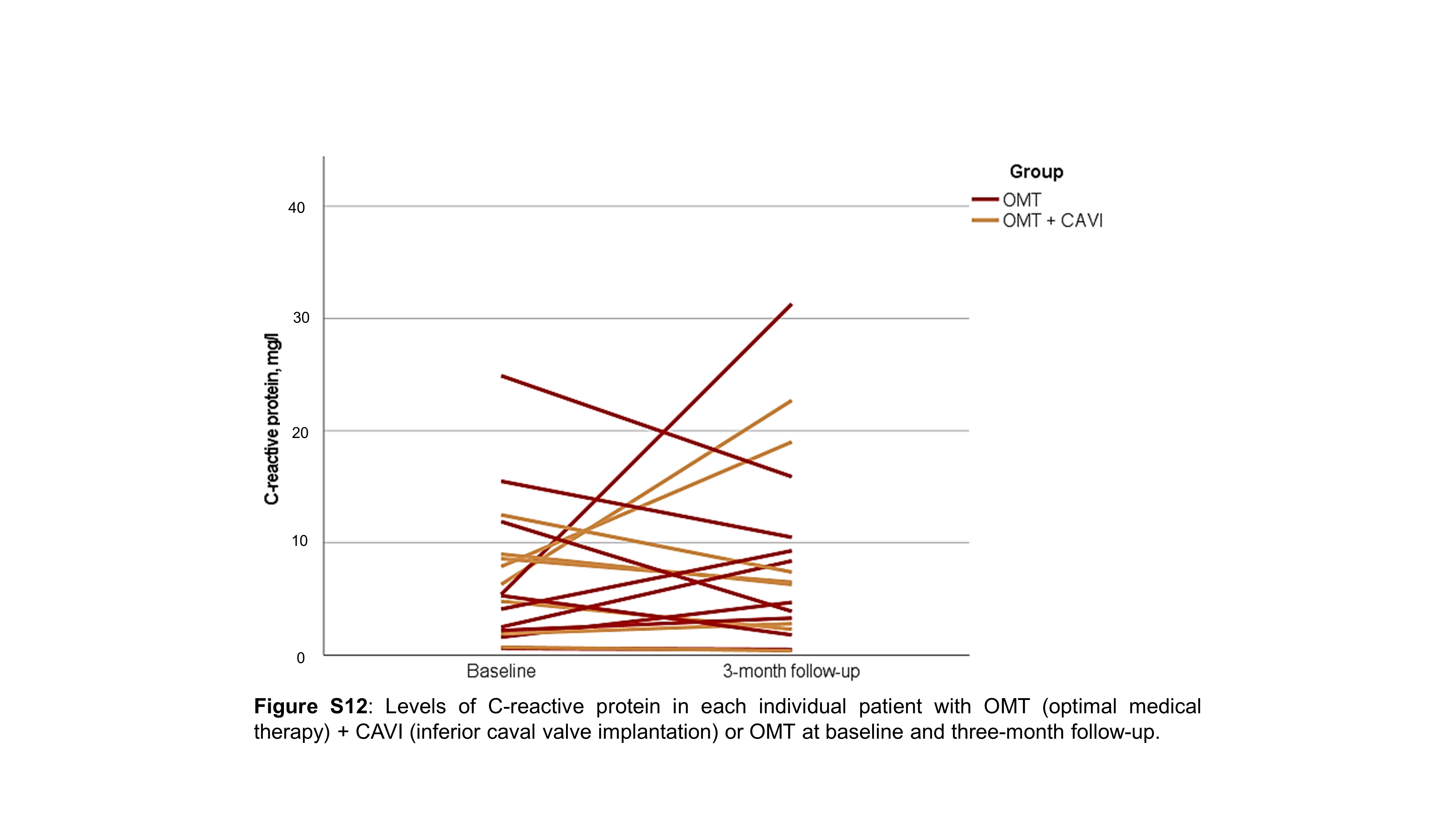

Supplement: Supplementary file 12 — Supplementary Material 12 [file 12872_2024_4044_MOESM12_ESM.tif]

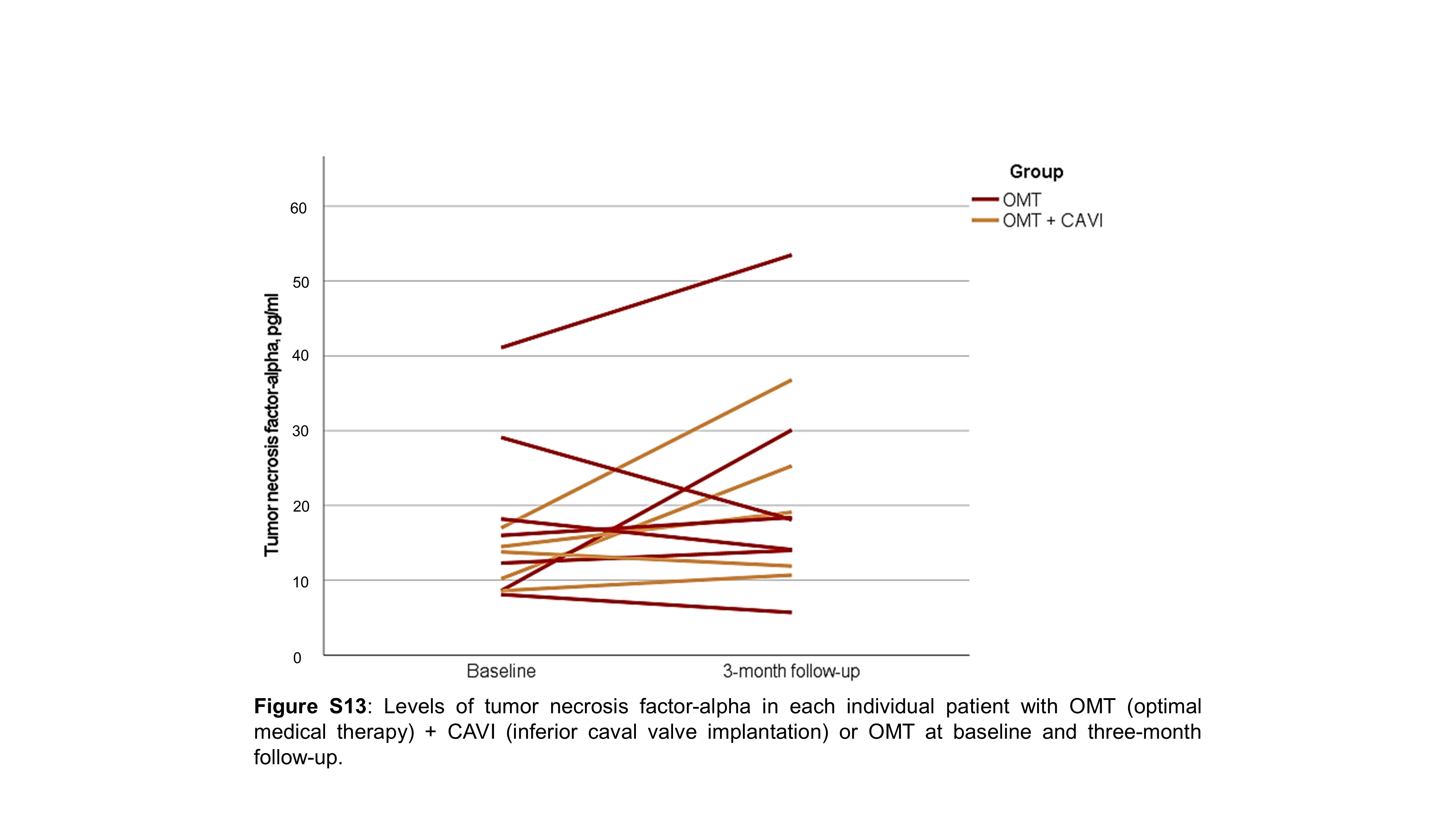

Supplement: Supplementary file 13 — Supplementary Material 13 [file 12872_2024_4044_MOESM13_ESM.tif]

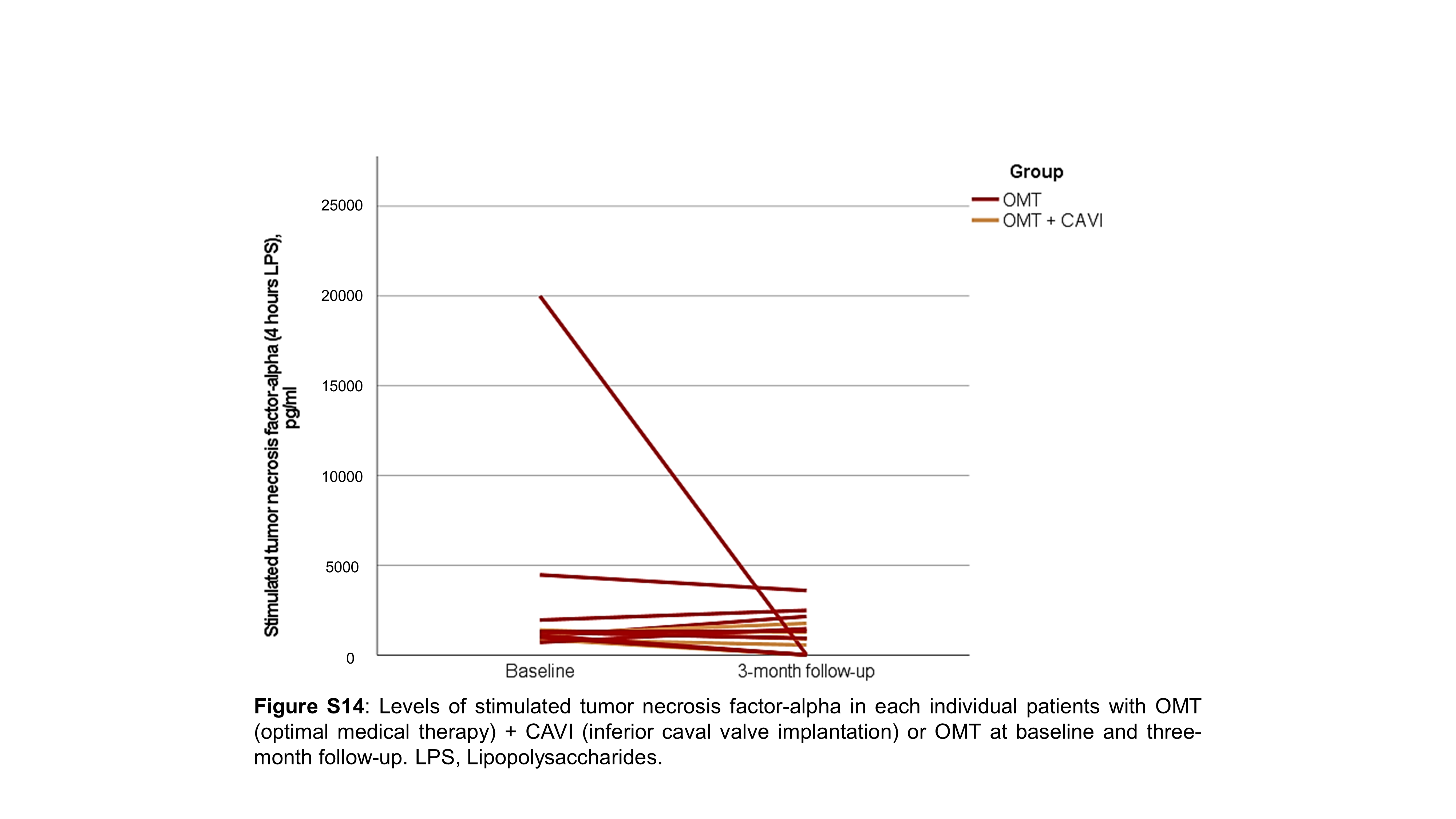

Supplement: Supplementary file 14 — Supplementary Material 14 [file 12872_2024_4044_MOESM14_ESM.tif]

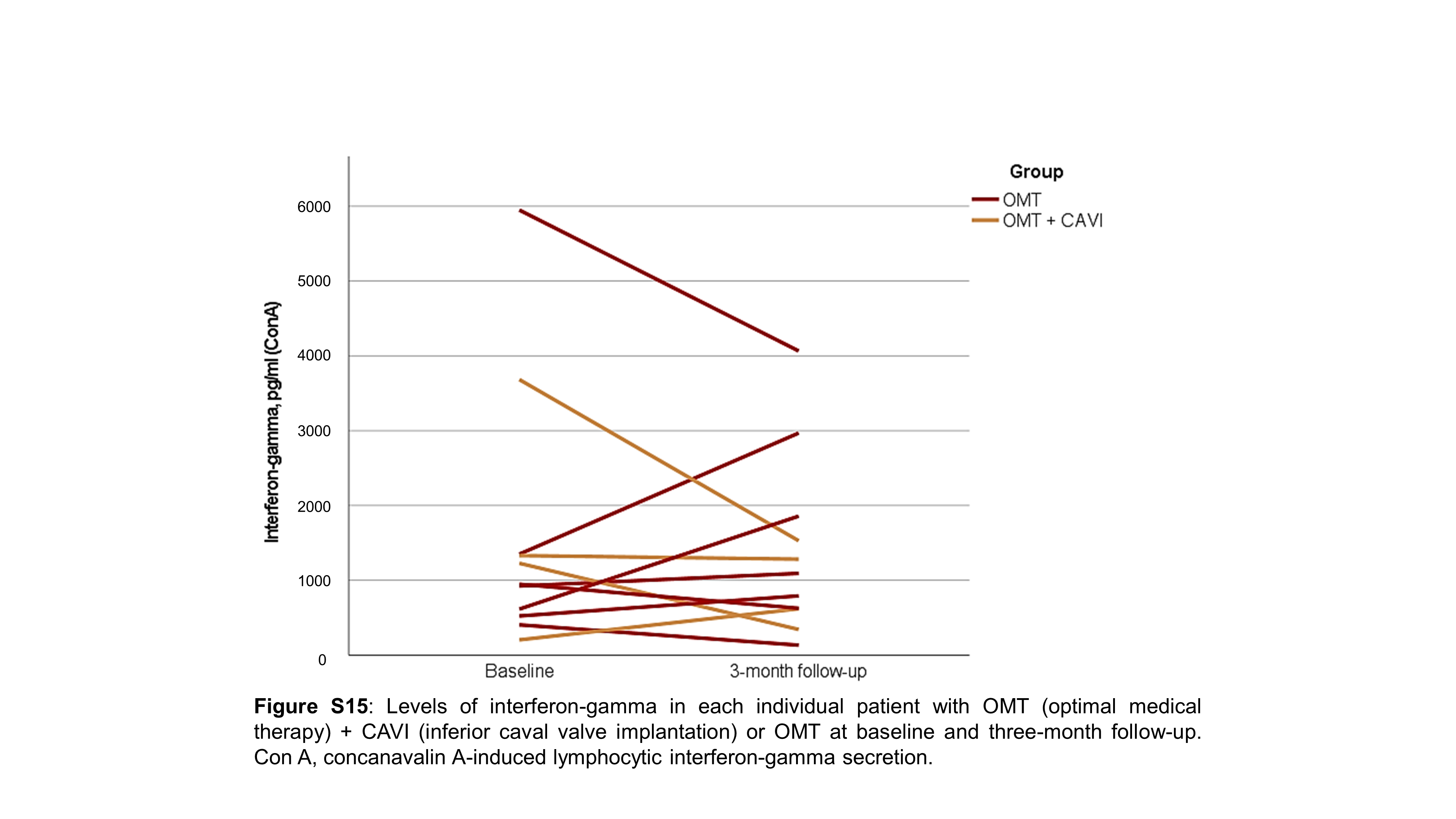

Supplement: Supplementary file 15 — Supplementary Material 15 [file 12872_2024_4044_MOESM15_ESM.tif]

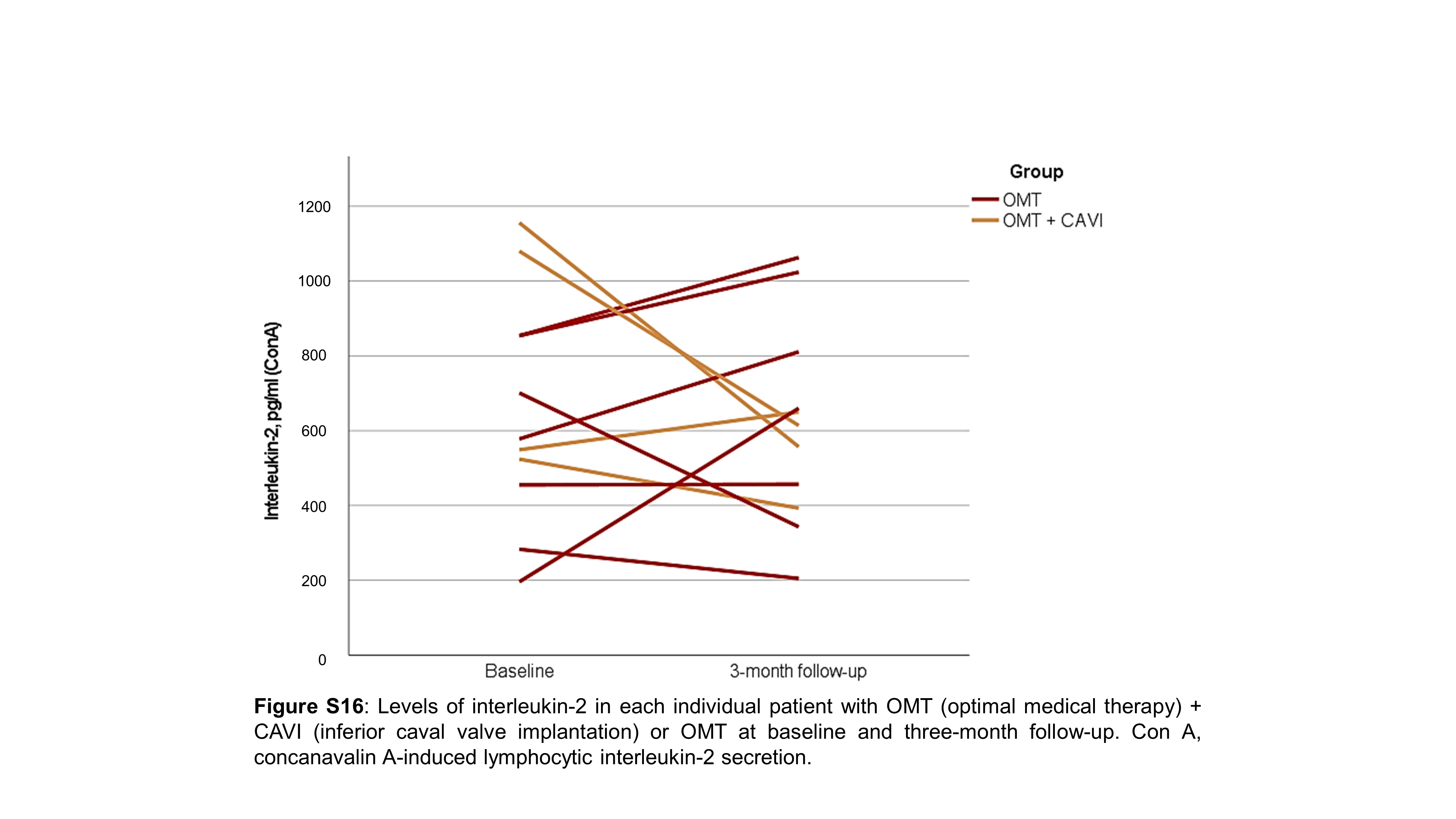

Supplement: Supplementary file 16 — Supplementary Material 16 [file 12872_2024_4044_MOESM16_ESM.tif]

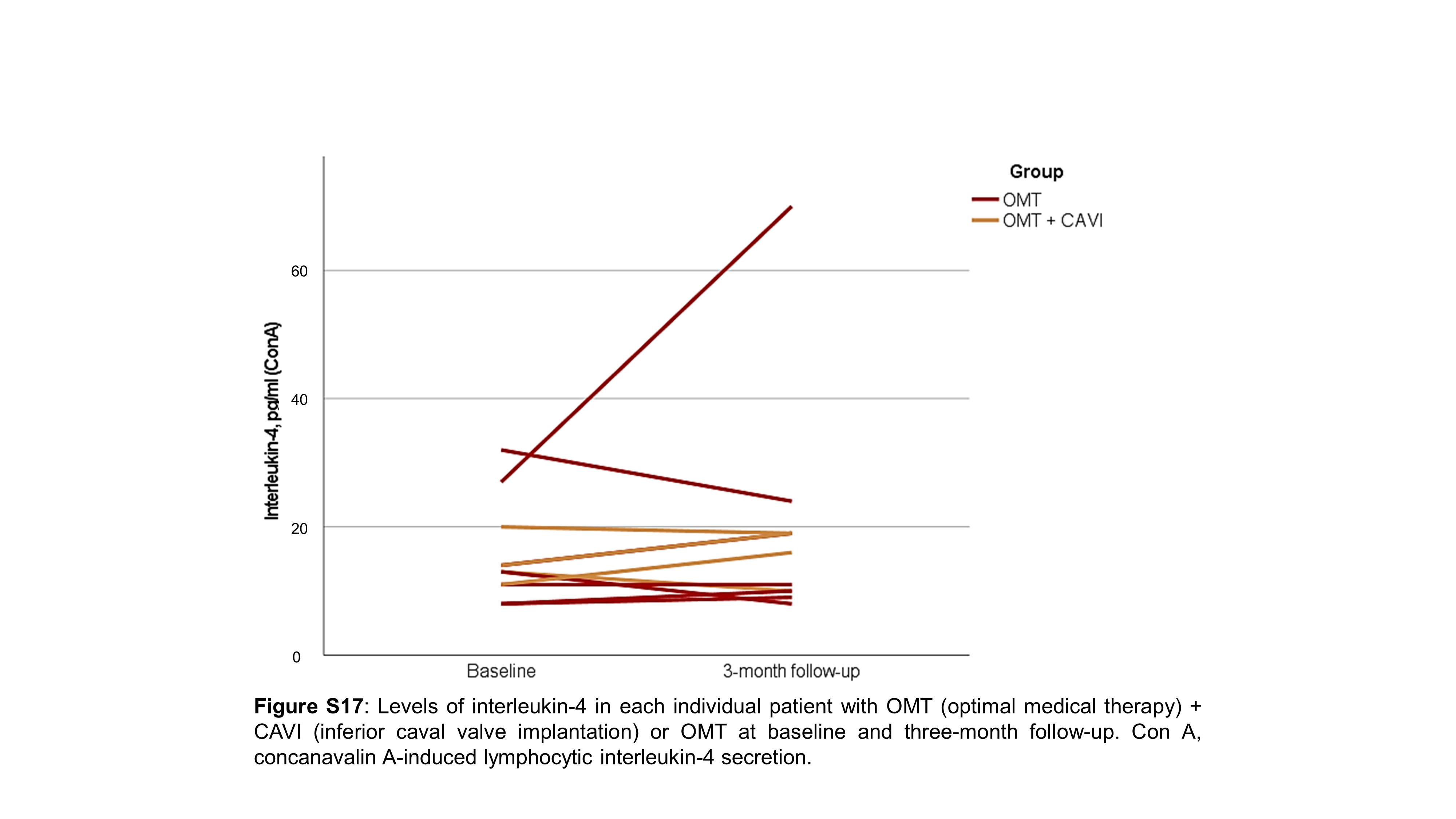

Supplement: Supplementary file 17 — Supplementary Material 17 [file 12872_2024_4044_MOESM17_ESM.tif]

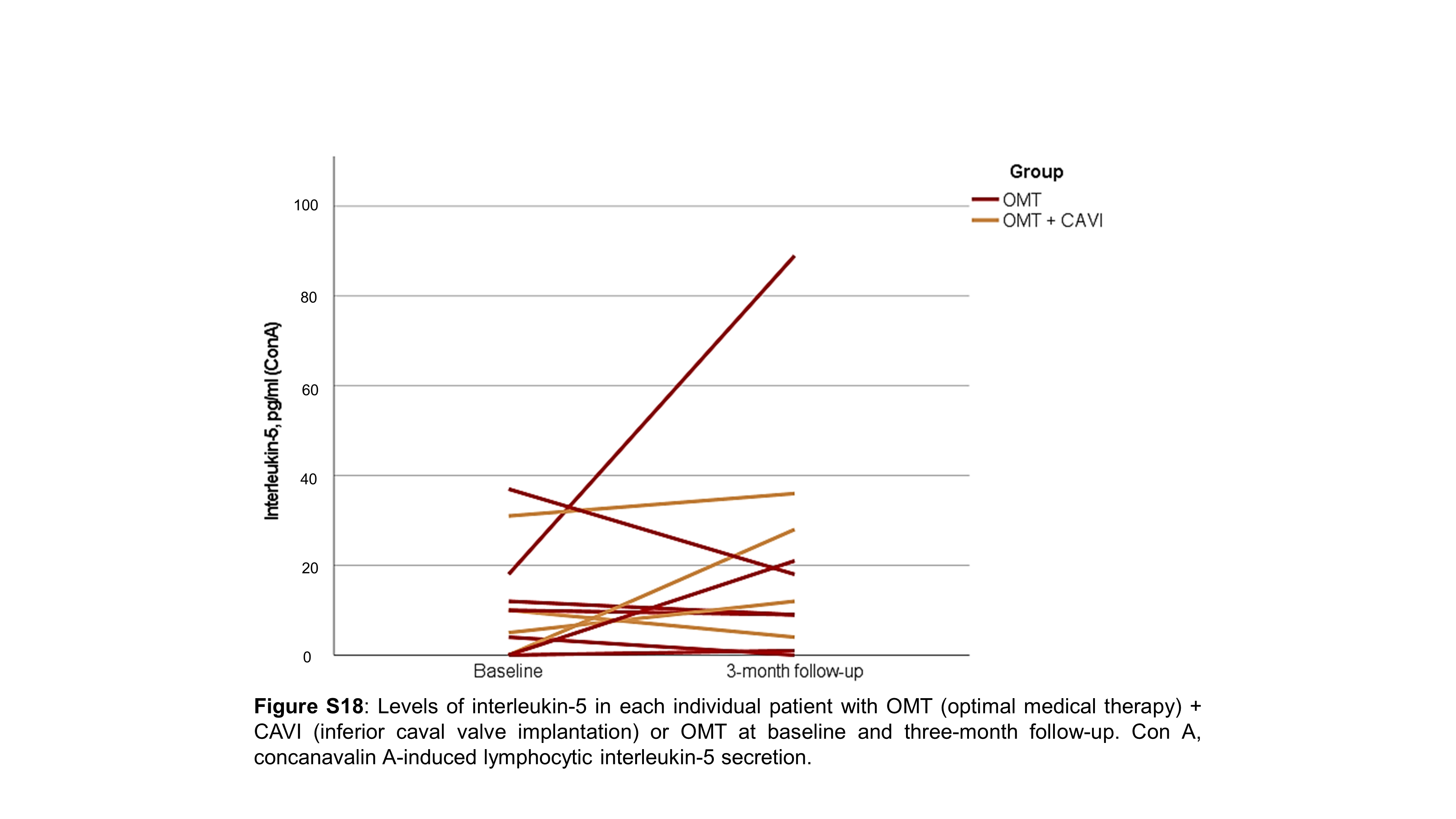

Supplement: Supplementary file 18 — Supplementary Material 18 [file 12872_2024_4044_MOESM18_ESM.tif]

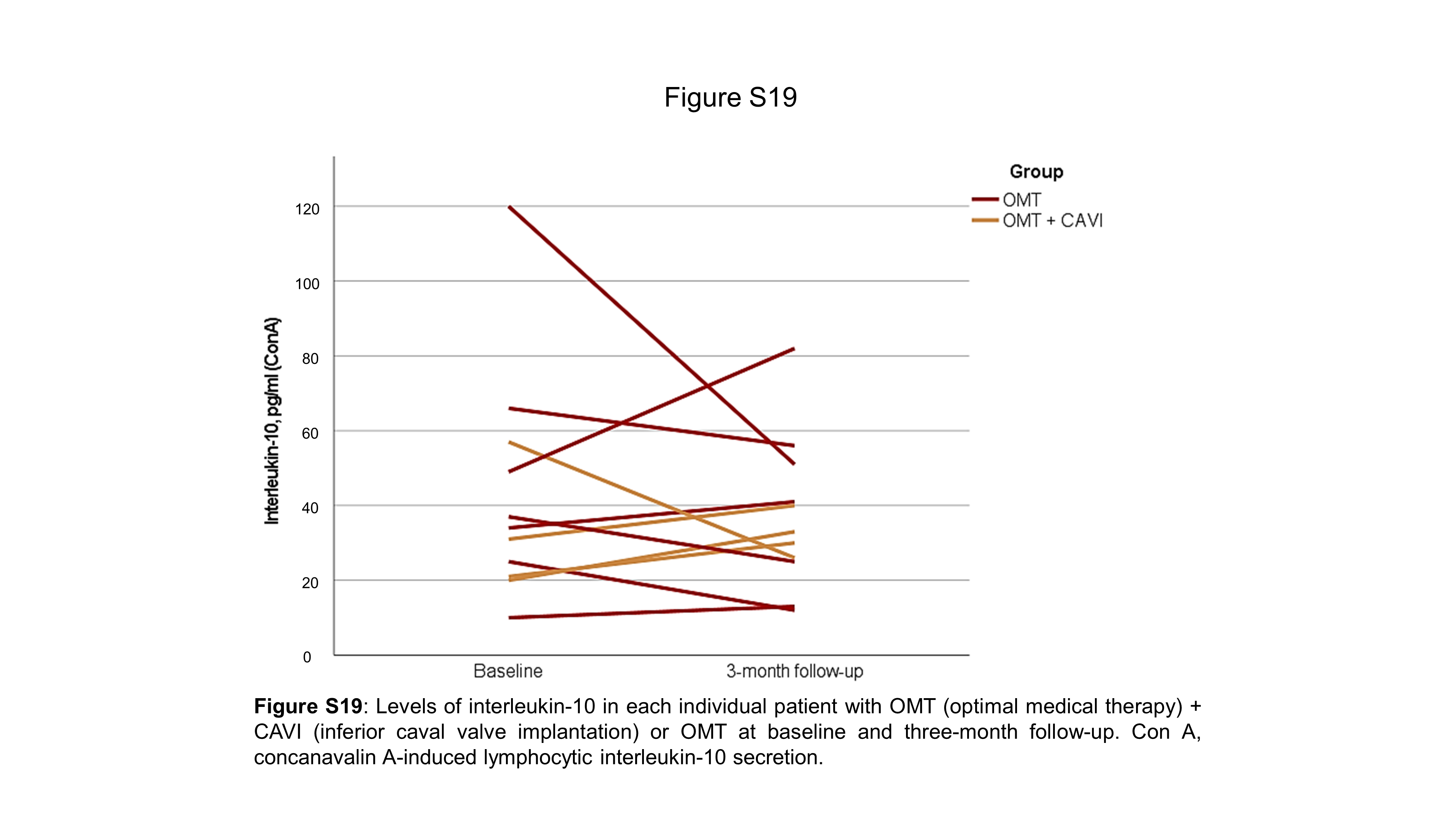

Supplement: Supplementary file 19 — Supplementary Material 19 [file 12872_2024_4044_MOESM19_ESM.tif]
