# Supplementary material for: Effect of inferior caval valve implantation on circulating immune cells and inflammatory mediators in severe tricuspid regurgitation
Source: BMC Cardiovasc Disord. 2024 Jul 18;24:373. doi: 10.1186/s12872-024-04044-1 (PMC11256587; doi:10.1186/s12872-024-04044-1)
Supplement: Supplementary file 20 — Supplementary Material 20 [file 12872_2024_4044_MOESM20_ESM.docx]

**Supplement**

**Effect of** **inferior caval valve implantation on** **circulating immune cells and inflammatory mediators in severe tricuspid regurgitation**

Isabel Mattig, MD^1,2,3,4*^; Bernd Hewing, MD^1,2,3,6*^; Fabian Knebel, MD^1,2,3,4,5^; Christian Meisel, MD^7^; Antje Ludwig^1,2^; Frank Konietschke^8^; Verena Stangl, MD^1,2,4^; Karl Stangl, MD^1,2,4^; Michael Laule, MD^1,2,4^; Henryk Dreger, MD^2,4,9^

^1^) Deutsches Herzzentrum der Charité, Department of Cardiology, Angiology and Intensive Care Medicine, Campus Charité Mitte, Berlin, Germany

^2^) Charité – Universitätsmedizin Berlin, corporate member of Freie Universität Berlin and Humboldt-Universität zu Berlin, Charitéplatz 1, 10117 Berlin, Germany

^3^) Berlin Institute of Health at Charité – Universitätsmedizin Berlin, BIH Biomedical Innovation Academy, Berlin, Germany

^4^) DZHK (German Centre for Cardiovascular Research), partner site Berlin, Germany

^5^) Sana Klinikum Lichtenberg, Innere Medizin II: Schwerpunkt Kardiologie, Berlin, Germany

^6^) Department of Cardiology III - Adult Congenital and Valvular Heart Disease, University Hospital Muenster, Muenster, Germany

^7^) Labor Berlin – Charité Vivantes Services GmbH, Berlin, Germany

^8^) Institute for Biometry and Clinical Epidemiology, Charité – Universitätsmedizin Berlin, corporate member of Freie Universität Berlin and Humboldt-Universität zu Berlin, Charitéplatz 1, 10117 Berlin, Germany.

^9^) Deutsches Herzzentrum der Charité, Department of Cardiology, Angiology and Intensive Care Medicine, Campus Virchow Klinikum, Berlin, Germany

*I. Mattig and B. Hewing contributed equally to this work.

**Tables:**

| **Table S1**: Circulating immune cells drawn from a peripheral vein of patients with optimal CAVI (*n*=7) at baseline and three-month follow-up. | | | | |
| --- | --- | --- | --- | --- |
|  | Baseline | 3 months | *p* for intragroup comparison | *z* value for intragroup comparison |
| Leukocytes (IQR), /nl | 5.7 (4.9-6.6) | 7.0 (5.1-7.5) | 0.128 | -1.521 |
| Lymphocytes (IQR), /nl | 1.1 (0.8-1.4) (*n*=4) | 1.0 (1.0-1.1) (*n*=4) | 0.465 | -0.730 |
| Neutrophils (IQR), /nl | 3.7 (3.1-4.5) (*n*=6) | 4.6 (2.5-5.6) (*n*=6) | 0.463 | -0.734 |
| Eosinophils (IQR), /nl | 0.2 (0.1-0.4) (*n*=4) | 0.2 (0.1-0.5) (*n*=4) | 1.000 | 0.000 |
| Basophils (IQR), /nl | 0.1 (0.0-0.7) (*n*=4) | 0.0 (0.0-0.1) (*n*=4) | 0.593 | -0.535 |
| Monocytes  (IQR), /nl | 0.5 (0.4-0.6) | 0.4 (0.4-0.5) (*n*=4) | 0.176 | -1.352 |
| CD4+ CD8+ cell ratio (IQR) | 4.9 (1.6-7.3) | 4.1 (1.7-5.7) | 0.310 | -1.014 |
| CD8- CD4- T cells (IQR), percent of T cells | 3.5 (1.0-5.1) | 3.7 (1.4-5.8) | 0.237 | -1.183 |
| CD8+ CD4+ T cells (IQR), percent of T cells | 1.4 (1.2-1.7) | 1.7 (1.1-2.1) | 0.735 | -0.338 |
| CD3+ T cells (IQR), percent of lymphocytes | 65.0 (37.0-76.0) | 72.0 (63.0-85.0) | 0.128 | -1.524 |
| CD4+ T cells (IQR), percent of lymphocytes | 45.0 (28.0-51.0) | 48.0 (45.0-64.0) | 0.395 | -0.851 |
| CD4+ T cells (IQR), percent of T cells | 71.6 (53.9-83.1) | 75.9 (58.1-80.4) | 0.735 | -0.338 |
| CD8+ T cells (IQR), percent of lymphocytes | 9.0 (6.0-27.0) | 12.0 (11.0-26.0) | 0.041 | -2.043 |
| CD8+ T cells (IQR), percent of T cells | 15.5 (14.0-35.9) | 18.7 (14.5-35.0) | 0.612 | -0.507 |
| CD28+ T cells (IQR), percent of CD8- T cells | 97.0 (88.5-99.0) (*n*=6) | 95.5 (87.8-99.0) (*n*=6) | 0.180 | -1.342 |
| CD28+ T cells (IQR), percent of CD8+ T cells | 62.0 (27.8-68.5) (*n*=6) | 59.5 (30.8-67.8) (*n*=6) | 0.916 | -0.105 |
| CD57+ T cells (IQR), percent of CD8- T cells | 4.0 (1.0-10.3) (*n*=6) | 6.0 (2.0-10.0) (*n*=6) | 0.129 | -1.518 |
| CD57+ T cells (IQR), percent of CD8+ T cells | 35.5 (23.5-49.3) (*n*=6) | 35.0 (26.8-51.0) (*n*=6) | 0.458 | -0.742 |
| HLA-DR+ T cells (IQR), percent of CD8- T cells | 9.0 (5.5-13.0) (*n*=6) | 9.5 (8.5-17.8) (*n*=6) | 0.498 | -0.677 |
| HLA-DR+ T cells (IQR), percent of CD8+ T cells | 18.5 (11.0-47.0) (*n*=6) | 27.5 (20.0-37.5) (*n*=6) | 0.600 | -0.524 |
| CD3+ CD8+ cells (IQR), percent of CD3+ cells | 20.2 (13.6-37.1) (*n*=5) | 23.6 (13.2-30.6) (*n*=5) | 0.225 | -1.214 |
| CD3+ CD8- cells (IQR), percent of CD3+ cells | 72.2 (56.6-80.9) (*n*=5) | 66.8 (62.0-81.6) (*n*=5) | 0.345 | -0.944 |
| naive CD45RA+ CCR7+ cells (IQR), percent of CD8+ cells | 3.4 (3.1-10.9) (*n*=5) | 4.3 (2.4-14.4) (*n*=5) | 0.893 | -0.135 |
| TEMRA CD45RA+ CCR7- cells ± SD, percent of CD8+ cells | 33.4 (23.1-59.0) (*n*=5) | 26.9 (23.2-57.7) (*n*=5) | 0.893 | -0.135 |
| Naive CD45+ CCR7+ cells (IQR), percent of CD4+ cells | 19.2 (10.0-21.6) (*n*=5) | 23.1 (7.6-25.1) (*n*=5) | 0.500 | -0.674 |
| TEMRA CD45RA+ CCR7- cells (IQR), percent of CD4+ cells | 1.0 (0.1-2.5) (*n*=5) | 1.5 (0.4-2.1) (*n*=5) | 0.893 | -0.135 |
| CD45RA+ cells (IQR), percent of CD4+ cells | 20.4 (10.5-23.5) (*n*=5) | 25.2 (8.9-26.1) (*n*=5) | 0.225 | -1.214 |
| CD45RA- cells (IQR), percent of CD4+ cells | 79.6 (76.5-89.5) (*n*=5) | 74.9 (73.9-91.1) (*n*=5) | 0.225 | -1.214 |
| CD25+ CD127- T regulatory cells (IQR), percent of CD4+ cells | 9.5 (8.8-9.8) (*n*=5) | 8.3 (6.3-9.1) (*n*=5) | 0.138 | -1.483 |
| CD19+ B cells (IQR), percent of lymphocytes | 10.0 (5.0-18.0) | 6.0 (4.0-13.0) | 0.041 | -2.047 |
| Natural killer cells (IQR), percent of lymphocytes | 14.0 (10.0-19.0) | 17.0 (11.0-24.0) | 0.499 | -0.676 |
| Continuous variables are shown as median and interquartile ranges (IQR) due to the distribution of parameters (uniform per variable). OMT, optimal medical therapy; CAVI, caval valve implantation; n, number of patients with laboratory measurements in case of missing data. | | | | |

| **Table S2**: Circulating inflammatory markers drawn from a peripheral vein of patients with optimal CAVI (*n*=7) at baseline and three-month follow-up. | | | | |
| --- | --- | --- | --- | --- |
|  | Baseline | 3 months | *p* for intragroup comparison | *z* value for intragroup comparison |
| Non-stimulated | | | | |
| C-reactive protein (IQR), mg/l | 7.9 (1.9-9.0) | 6.3 (2.3-7.4) | 0.398 | -0.845 |
| Tumor necrosis factor-alpha (IQR), pg/ml | 13.8 (9.4-15.8) (*n*=5) | 19.1 (11.3-31.1) (*n*=5) | 0.080 | -1.753 |
| Stimulated | | | | |
| Stimulated tumor necrosis factor-alpha (4 hours LPS) (IQR), pg/ml | 1180.0 (869.0-1353.0) (*n*=5) | 909.0 (286.4-1523.0) (*n*=5) | 0.345 | -0.944 |
| Interferon-gamma (IQR), pg/ml (ConA) | 1279.5 (460.0-3095.8) (*n*=4) | 951.0 (412.0-1467.5) (*n*=4) | 0.273 | -1.095 |
| Interleukin-2 (IQR), pg/ml (ConA) | 814.5 (530.3-1137.0) (*n*=4) | 585.5 (434.0-641.0) (*n*=4) | 0.144 | -1.461 |
| Interleukin-4 (IQR), pg/ml (ConA) | 13.5 (11.5-18.5) (*n*=4) | 17.5 (11.5-19.0) (*n*=4) | 0.461 | -0.736 |
| Interleukin-5 (IQR), pg/ml (ConA) | 7.5 (1.3-25.8) (*n*=4) | 20.0 (6.0-34.0) (*n*=4) | 0.273 | -1.095 |
| Interleukin-10 (IQR), pg/ml (ConA) | 26.0 (20.3-50.5) (*n*=4) | 31.5 (27.0-38.3) (*n*=4) | 0.713 | -0.368 |
| Continuous variables are shown as median and interquartile ranges (IQR) due to the distribution of parameters (uniform per variable). OMT, optimal medical therapy; CAVI, caval valve implantation; *n*, number of patients with laboratory measurements in case of missing data. LPS, Lipopolysaccharides; Con A, concanavalin A-induced lymphocytic IFN-γ, TNF-α, IL-2, IL-4, IL-5, and IL-10 secretion. | | | | |
